# Correction: *Childhood maltreatment and biomarkers for cardiometabolic disease in mid-adulthood in a prospective British birth cohort: associations and potential explanations*

**DOI:** 10.1136/bmjopen-2018-024079corr1

**Published:** 2019-06-19

Li L, Pinto Pereira SM, Power C. Childhood maltreatment and biomarkers for cardiometabolic disease in mid-adulthood in a prospective British birth cohort: associations and potential explanations *BMJ Open* 2019;**9:**e024079. doi: 10.1136/bmjopen-2018-024079

This article was previously published with an error.

The correct copyright information is as follows:

