# Supplementary material for: Childhood maltreatment and biomarkers for cardiometabolic disease in mid-adulthood in a prospective British birth cohort: associations and potential explanations
Source: BMJ Open. 2019 Mar 23;9(3):e024079. doi: 10.1136/bmjopen-2018-024079 (PMC6475361; doi:10.1136/bmjopen-2018-024079)
Supplement: Supplementary data [file bmjopen-2018-024079supp001.pdf]

Supplemental Table S1: Definition of child maltreatment and representative variables from the 1958 British birth cohort.

	Definition(44)	1958 British birth cohort variables*	Reference age	Age collected (Ascertainment method¥)
Neglect (prospective)‡	Failure to meet a child's basic physical, emotional, medical/dental, or education need; failure to provide adequate nutrition, hygiene, or shelter; or failure to ensure a child's safety	constructed from:		
		- child looks undernourished, scruffy or dirty	7y, 11y	7y, 11y(T)
		- hardly ever takes outings with mother	7y, 11y	7y, 11y(P)
		- hardly ever takes outings with father	7y, 11y	7y, 11y(P)
		- mother has little interest in education	7y, 11y	7y, 11y(T)
		- father has little interest in education	7y, 11y	7y, 11y(T)
Sexual abuse	Any completed or attempted sexual act, sexual contact, or non-contact sexual interaction with a child by a caregiver.	- I was sexually abused by a parent	0-16y	45y(S)
Physical abuse	Intentional use of physical force or implements against a child that results in, or has the potential to result in, physical injury.	- I was physically abused by a parent – punched, kicked or hit or beaten with an object, or needed medical treatment	0-16y	45y(S)
Psychological abuse	Intentional behaviour that conveys to a child that h/she is worthless, flawed, unloved, unwanted, endangered, or valued only in meeting another's needs. *UK definition includes harmful (unintentional) parent-child interactions: 'the persistent emotional ill-treatment of a child such as to cause severe and persistent adverse effects on the child's emotional development'*	- I was verbally abused by a parent<br>- I suffered humiliation, ridicule, bullying or mental cruelty from a parent	0-16y	45y(S)

*for retrospective reports at 45y, information was obtained via CASI (direct computer data entry) derived from the Personality and Total Health Through Life Project (50), details of which are provided elsewhere(25). Participants were instructed: "The following are statements about your childhood. For each, please say whether the statement applies to you." Response options were: "Yes", "No", "Can't say".

¥ (S): self-report; (T): teacher-report; (P): parent-report

‡neglect (prospective) defined as ≥2 items at either 7 or 11y (note at each age, if one or two items were missing we used the sum of the remaining 4 or 3 items respectively; if >2 items were missing, we treated neglect as missing)

Supplemental Table S2 Associations (mean difference or OR) between childhood maltreatment and cardiometabolic biomarkers at 45y – adjusted for lifestyle factors individually and jointly

**Mean difference (95%CI)**‡	Model 2	+adult lifestyle factors˜	+smoking only	+alcohol consumption only	+physical activity only
**Neglect**					
BMI (kg/m^2)	**0.53(0.23,0.83)**	**0.50(0.20,0.80)**	**0.60(0.31,0.90)**	**0.42(0.13,0.72)**	**0.51(0.21,0.81)**
Waist circumference (cm)	**1.23(0.51,1.96)**	**1.09(0.37,1.81)**	**1.30(0.58,2.03)**	**1.03(0.30,1.75)**	**1.18(0.46,1.90)**
HDL-c (mmol/L) (females)	**-0.05(-0.08,-0.01)**	-0.02(-0.06,0.02)	-0.03(-0.07,0.004)	-0.03(-0.07,0.004)	-0.05(-0.08,0.01)
Triglycerides† %	**3.9(0.3,7.5)**	2.4(-1.2,6.0)	2.8(-0.8,6.4)	3.5(-0.1,7.1)	**3.8(0.2,7.4)**
HbA_{1c}(%)† %	**1.2(0.4,2.0)**	0.7(-0.1,1.5)	**0.9(0.1,1.7)**	**1.0(0.2,1.8)**	**1.2(0.4,2.0)**
**Physical abuse**					
BMI (kg/m^2)	**0.72(0.28,1.16)**	**0.79(0.36,1.22)**	**0.83(0.39,1.27)**	**0.65(0.21,1.08)**	**0.73(0.30,1.17)**
Waist circumference (cm)	**1.29(0.23,2.35)**	**1.32(0.27,2.37)**	**1.38(0.32,2.44)**	**1.13(0.09,2.19)**	**1.32(0.27,2.37)**
HDL-c (mmol/L) (females)	**-0.06(-0.12,-0.01)**	-0.04(-0.09,0.02)	-0.05(-0.10,0.01)	**-0.05(-0.12,-0.01)**	**-0.06(-0.12,-0.01)**
HbA_{1c} (%) (males)† %	**2.5(0.7,4.3)**	**1.9(0.1,3.7)**	**1.9(0.1,3.7)**	**2.3(0.5,4.1)**	**2.5(0.7,4.3)**
**Sexual abuse**					
HbA_{1c}(%)† %	**2.4(0.0,4.8)**	1.2(-1.2,3.6)	1.6(-0.8,4.0)	1.9(-0.5,4.3)	2.2(-0..2,4.6)
**Psychological abuse**					
HDL-c (mmol/L)	**-0.04(-0.07,-0.01)**	-0.02(-0.05,0.004)	**-0.03(-0.06,-0.005)**	-0.03(-0.06,0.001)	**-0.04(-0.07,-0.013)**
**OR (95%CI) for elevated levels**$					
**Neglect**					
General obesity	**1.16(1.02,1.32)**	1.13(0.99,1.29)	**1.18(1.03,1.34)**	1.11(0.98,1.27)	**1.15(1.01,1.31)**
Central obesity	**1.15(1.02,1.30)**	**1.13(1.00,1.27)**	**1.16(1.03,1.31)**	**1.12(0.99,1.26)**	**1.14(1.01,1.29)**
**Physical abuse**					
General obesity	**1.36(1.13,1.64)**	**1.38(1.14,1.66)**	**1.39(1.15,1.68)**	**1.32(1.10,1.60)**	**1.37(1.14,1.65)**
Central obesity	**1.38(1.16,1.65)**	**1.39(1.17,1.66)**	**1.40(1.18,1.67)**	**1.36(1.14,1.62)**	**1.39(1.17,1.66)**
LDL-c	**1.25(1.00,1.56)**	1.16(0.93,1.46)	1.16(0.93,1.46)	1.24(0.99,1.55)	**1.25(1.00,1.57)**
**Sexual abuse**					
LDL-c	1.41(0.89,2.23)	1.26(0.79,2.00)	1.28(0.80,2.03)	1.39(0.87,2.20)	1.40(0.88,2.22)
**Psychological abuse**					
Triglycerides	**1.21(1.02,1.44)**	1.18(0.99,1.40)	1.18(0.99,1.40)	**1.20(1.01,1.43)**	**1.22(1.03,1.45)**

All cardiometabolic markers (continuous measures) were adjusted for medication. For binary outcomes, those on medication were in risk groups. Estimates that reached significance with *P*<0.05 were bold-faced.

Model 2 includes all in model 1, plus self-reported family history of diabetes (for HbA_{1c}, diabetes) and early-life factors (birthweight for gestational age, social class at birth, housing tenure and crowding at 7y).

†log transformed and converted to %

‡all cardiometabolic markers (continuous measures) were adjusted for medication. For binary outcomes (dyslipidemia), those on medication were in risk groups

˜shown in Table 3 as model 5: includes all in model 2, plus lifestyle factors at 42y (smoking status, alcohol consumption and physical activity).

Supplemental Table S3 Associations (mean difference or OR) for multiple types of maltreatment and cardiometabolic markers at 45y: unadjusted and adjusted for early-life factors

	**Number of maltreatments**							
	**0**	**1**		**2**		**≥3**		Per increase[#]
**Mean difference (95%CI)‡**		Model 1	Model 2	Model 1	Model 2	Model 1	Model 2	
BMI (kg/m^2)	-	**0.74(0.47,1.01)**	**0.47(0.19,0.74)**	**0.87(0.35,1.38)**	**0.66(0.15,1.17)**	**0.91(0.09,1.74)**	0.65(-0.18,1.47)	**0.33(0.17,0.49)**
Waist circumference (cm)								
males	-	0.74(-0.10,1.58)	0.29(-0.56,1.14)	1.13(-0.60,2.86)	0.88(-0.83,2.60)	-0.09(-3.20,3.02)	-0.46(-3.55,2.63)	0.23(-0.33,0.79)
females	-	**2.74(1.74,3.73)**	**2.19(1.17,3.21)**	**2.47(0.63,4.30)**	**2.00(0.18,3.82)**	2.32(-0.26,4.90)	1.84(-0.75,4.43)	**1.14(0.57,1.71)**
Blood pressure								
SBP mmHg	-	**1.22(0.38,2.06)**	0.70(-0.18,1.57)	-0.34(-1.96,1.28)	-0.76(-2.37,0.86)	-0.81(-3.35,1.73)	-1.53(-4.07,1.01)	-0.10(-0.61,0.41)
DBP mmHg	-	**1.01(0.43,1.59)**	**0.67(0.08,1.27)**	0.42(-0.69,1.53)	0.15(-0.96,1.25)	0.01(-1.75,1.77)	-0.45(-2.21,1.31)	0.18(-0.17,0.53)
Blood lipids								
Total cholesterol mmol/L	-	**0.06(0,0.13)**	0.05(-0.02,0.11)	0.01(-0.12,0.14)	0.01(-0.12,0.14)	0.08(-0.12,0.28)	0.07(-0.14,0.27)	0.02(-0.02,0.06)
**HDL-c mmol/L**								
**males**	-	-0.02(-0.05,0.01)	**-0.003(-0.031,0.025)**	-0.03(-0.09,0.02)	-0.02(-0.08,0.04)	-0.04(-0.15,0.07)	-0.03(-0.14,0.08)	-0.01(-0.03,0.01)
**females**	-	-0.08(-0.11,0.05)	**-0.05(-0.09,-0.02)**	-0.09(-0.15,0.02)	**-0.06(-0.13,0.0)**	-0.12(-0.21,0.03)	**-0.09(-0.18,0.0)**	**-0.04(-0.06,-0.02)**
LDL-c	-	**0.07(0.01,0.13)**	**0.06(0,0.11)**	0.04(-0.08,0.15)	0.02(-0.09,0.14)	0.09(-0.08,0.27)	0.08(-0.10,0.25)	0.03(-0.01,0.07)
Triglycerides† %	-	**6.3(2.9,9.7)**	**3.2(0,6.6)**	4.5(-1.9,10.9)	1.9(-4.5,8.3)	**10.8(0.6,21.0)**	7.4(-2.6,17.4)	**2.2(0.0,4.4)**
HbA_{1c} † %	-	**1.8(1,2.6)**	**1.4(0.6,2.2)**	**2.1(0.7,3.5)**	**1.7(0.3,3.1)**	**1.0(-1.4,3.4)**	0.5(-1.9,2.9)	**0.8(0.4,1.3)**
**OR (95%CI) for elevated levels$**								
General obesity	-	**1.29(1.15,1.45)**	**1.19(1.03,1.37)**	**1.39(1.11,1.73)**	**1.33(1.03,1.72)**	**1.45(1.03,2.05)**	1.37(0.93,2.00)	**1.13(1.05,1.21)**
Central obesity	-	**1.25(1.12,1.39)**	**1.16(1.04,1.29)**	**1.39(1.13,1.71)**	**1.32(1.07,1.62)**	1.26(0.91,1.74)	1.18(0.85,1.64)	**1.12(1.05,1.20)**
Hypertension	-	**1.18(1.06,1.34)**	1.11(0.98,1.26)	1.02(0.81,1.30)	0.97(0.76,1.23)	0.91(0.61,1.36)	0.84(0.56,1.25)	1.01(0.94,1.09)
HDL-c	-	**1.29(1.11,1.50)**	1.14(0.97,1.33)	**1.56(1.18,2.07)**	**1.41(1.06,1.87)**	1.17(0.74,1.85)	1.02(0.65,1.62)	**1.11(1.01,1.21)**
LDL-c	-	1.10(0.96,1.25)	1.06(0.92,1.22)	1.17(0.89,1.54)	1.14(0.86,1.50)	1.16(0.76,1.78)	1.12(0.73,1.72)	1.05(0.97,1.15)
Triglycerides	-	**1.24(1.09,1.40)**	**1.14(1.00,1.30)**	1.24(0.96,1.59)	1.16(0.90,1.50)	1.41(0.94,2.10)	1.30(0.87,1.94)	**1.10(1.01,1.20)**
HbA_{1c}	-	**1.47(1.15,1.87)**	**1.31(1.01,1.69)**	1.47(0.89,2.43)	1.33(0.80,2.21)	1.30(0.54,3.18)	1.12(0.46,2.74)	1.16(0.99,1.36)
Metabolic syndrome	-	**1.26(1.03,1.54)**	1.15(0.95,1.39)	1.37(0.94,1.99)	1.27(0.87,1.83)	1.15(0.61,2.16)	1.06(0.57,1.96)	1.10(0.96,1.26)

NB: analyses are for genders combined except where p≤0.05 for gender*maltreatment interaction where analyses are for males and females separately. Estimates that reached significance with *P*<0.05 were bold-faced.

[#] per increase based on Model 2

Model 1 includes gender and factors affecting measurement (measured room temperature for BP; month of examination, time of day of blood collection, postal delay of blood sample, time since last meal for lipids and HbA_{1c}; for females oral contraception and HRT for all outcomes). All factors were included for metabolic syndrome

Model 2 includes all in model 1, plus self-reported family history of diabetes (for HbA_{1c}, diabetes) and early-life factors, including birthweight for gestational age, social class at birth, housing tenure and crowding at 7y
†log transformed and converted to % [NB: for HbA_{1c} the parameters are % of the units (%)]
‡all cardiometabolic markers (continuous measures) were adjusted for medication. For binary outcomes (hypertension, dyslipidemia, or T2 diabetes), those on medication were in risk groups
$details of risk groups in **Table 1**
