# Supplementary material for: Childhood maltreatment and biomarkers for cardiometabolic disease in mid-adulthood in a prospective British birth cohort: associations and potential explanations
Source: BMJ Open. 2019 Mar 23;9(3):e024079. doi: 10.1136/bmjopen-2018-024079 (PMC6475361; doi:10.1136/bmjopen-2018-024079)
Supplement: Author's manuscript [file bmjopen-2018-024079.draft_revisions.pdf]

BMJ Open

BMJ Open is committed to open peer review. As part of this commitment we make the peer review history of every article we publish publicly available.

When an article is published we post the peer reviewers' comments and the authors' responses online. We also post the versions of the paper that were used during peer review. These are the versions that the peer review comments apply to.

The versions of the paper that follow are the versions that were submitted during the peer review process. They are not the versions of record or the final published versions. They should not be cited or distributed as the published version of this manuscript.

BMJ Open is an open access journal and the full, final, typeset and author-corrected version of record of the manuscript is available on our site with no access controls, subscription charges or pay-per-view fees (http://bmjopen.bmj.com).

If you have any questions on BMJ Open's open peer review process please email info.bmjopen@bmj.com

BMJ Open

Childhood maltreatment and biomarkers for cardiometabolic disease in mid-adulthood in a prospective birth cohort: associations and potential explanations

Journal:	*BMJ Open*
Manuscript ID	bmjopen-2018-024079
Article Type:	Research
Date Submitted by the Author:	08-May-2018

Complete List of Authors:	Li, Leah; UCL Institute of Child Health, Population Policy and Practice Programme<br>Pinto Pereira, Snehal; University College London Institute of Child Health, Population, Policy and Practice<br>Power, Christine

Keywords:	child maltreatment, child abuse, child neglect, cardiometabolic biomarkers, cohort study

SCHOLARONE™ Manuscripts

For peer review only

TITLE PAGE

Childhood maltreatment and biomarkers for cardiometabolic disease in mid-adulthood in a prospective birth cohort: associations and potential explanations

Leah Li (leah.li@ucl.ac.uk)*, Snehal M Pinto Pereira (snehal.pereira@ucl.ac.uk), Chris Power (christine.power@ucl.ac.uk)

Population, Policy, and Practice Programme, University College London Great Ormond Street Institute of Child Health, 30 Guilford Street, London WC1N 1EH, UK.

*** Corresponding author**

For peer review only

For peer review only - http://bmjopen.bmj.com/site/about/guidelines.xhtml

Abstract

Objectives: Research on associations between childhood maltreatment and adult cardiometabolic disease risk is sparse. We aimed to investigate associations between different forms of child maltreatment and mid-adult cardiometabolic markers and potential intermediaries for the associations.

Setting: 1958 British birth cohort.

Participants: Approximately 9 000 cohort members with data on cardiometabolic markers.

Outcomes: Adult (45y) cardiometabolic markers (blood pressure, lipids, glycated haemoglobin (HbA_{1c})). The associations of childhood neglect and abuse (physical, sexual, psychological) with cardiometabolic markers were examined adjusting for: (1) early-life covariates, (2) potential intermediary factors.

Results: Seventeen percent of participants were identified as neglected, 6%, 1.6%, and 10% as experiencing physical, sexual and psychological abuse respectively. Childhood neglect and physical abuse were associated with high BMI and waist circumference when adjusting for early-life covariates (e.g. for physical abuse the OR of central obesity was 1.38 (95% CI: 1.16, 1.65)) and with blood lipids and HbA_{1c} (e.g. for physical abuse the OR of high LDL-c was 1.25 (1.00, 1.56), HbA_{1c} was raised by 2.5 (0.7, 4.3)% (in males) and HDL-c was lower by 0.06 (0.01, 0.12) mmol/L (in females)). Associations for sexual abuse were similar to those for physical abuse but 95% CIs were wide. For psychological abuse the adjusted OR for elevated triglycerides was 1.21 (1.02, 1.44) and HDL-c was lower by 0.04 (0.01, 0.07) mmol/L. Maltreatments were not associated with raised blood pressure. In analyses of potential intermediary factors, several associations attenuated after adjustment for adult lifestyles and child-to-adult BMI.

Conclusions: Childhood maltreatments, particularly neglect and physical abuse, were associated with greater adiposity and poorer lipid and HbA_{1c} profiles decades later in adulthood. Associations were modest, but independent of early-life factors linked to these outcomes. Findings implicate adult lifestyles as an important intermediary factor.

Key words: child maltreatment, child abuse, child neglect, cardiometabolic biomarkers, cohort study

For peer review only - http://bmjopen.bmj.com/site/about/guidelines.xhtml

Strengths and limitations of this study

- Data were from a population-based cohort followed-up over five decades and included information on a range of adult cardiometabolic measures, childhood maltreatment (neglect and abuse), childhood covariates, and potential adult intermediary factors.
- Sample reductions due to missing information were addressed using multiple imputation.
- Study power for sexual abuse may be inadequate for detecting associations due to low prevalence.

For peer review only

For peer review only - http://bmjopen.bmj.com/site/about/guidelines.xhtml

Background

There is now evidence from several studies showing links between early-life adversities and later cardiovascular disease (CVD) incidence and mortality (1-3), including some with measures of child maltreatment (neglect and abuse) (4-7). In one example, the US adverse childhood experiences (ACE) study, reported child neglect or abuse was found to be associated with increased risk of self-reported ischemic heart disease (OR=1.3 to 1.7 for specific forms of maltreatment) with the highest risk observed for multiple ACEs (OR=3.6 (95% CI: 2.4, 5.3) for those with ≥7 vs 0) (4). Likewise, a recent US study found that those reporting ≥7 ACEs had a greater than threefold risk (OR=3.8 (1.9, 7.8)) of self-reported coronary heart disease compared to those reporting none and an elevated risk of diabetes (OR=1.4 (1.0, 2.1)) (5); and using self-reported childhood family psychosocial environment, a 1-unit increase in risky family score (range 0-21) was found to be associated with 1.0% higher 10-year coronary heart disease risk (using the Framingham algorithm) among 33-45 year-olds in the Coronary Artery Risk Development in Young Adults Study (6). Mortality follow-up studies have shown self-reported child abuse to predict mortality from all-causes in the US (8), whilst those with severe (vs. no) physical abuse were found to have increased risk for CVD events (hazard ratio 1.46 (1.11, 1.92)) in the Nurses Health Study. Accordingly, a scientific statement from the American Heart Association identified severe childhood adversities such as physical and sexual abuse as emerging independent risk factors for incident ischemic heart disease among women (9) .

However, much remains unknown about associations between child maltreatment and CVD risk, including whether associations are specific to different forms of maltreatment, whether neglect and abuse contribute to CVD risk independent of other early-life factors (such as low birthweight) linked to CVD and also, what the likely explanations or mechanisms are for any associations. Relevant to the latter is the growing evidence-base on child maltreatment and CVD risk factors, which provides important clues on possible intermediary pathways to cardiovascular events. Investigation of a range of cardiometabolic biomarkers would be informative, but studies to date typically focus solely on blood pressure (BP) (10-13) or obesity (14), and rarely include blood lipids (6;15;16) or glucose metabolism (17-19). Study findings are not always consistent: for example, some (10) but not all (11)

For peer review only - http://bmjopen.bmj.com/site/about/guidelines.xhtml

show associations of physical or sexual abuse with increased risk of raised BP. Differences in study design may account for such discrepancies. In particular, it is important to account for other early-life factors, such as low birthweight (1) and socio-economic position (SEP) (2) linked to these outcomes. Moreover, the literature mostly focuses on abuse (10;11;18;20) despite childhood neglect being associated with increased adult body mass index (BMI) (21), a risk factor for cardiometabolic disease, and when neglect is included it is often a component of a maltreatment score (4;17), or combined neglect/abuse measure (22). Combined measures may be useful to demonstrate cumulative effects but potentially obscure important differences that provide insights into mechanisms. Thus, a recent overview of long-term health consequences of non-sexual maltreatment concluded that research is required to confirm relationships between all forms of child maltreatment, including neglect, and later chronic disease (3).

With respect to explanations, pathways or mechanisms, notwithstanding a few exceptions (20), research is scarce on factors underlying the associations between forms of child maltreatment and biomarkers for adult cardiometabolic disease. Several potential intermediary factors can be postulated. First, associations may be due to greater BMI gains (20;23) or obesity (10;24) among maltreated groups. Second, child maltreatment may influence adult SEP (25), e.g. via poorer education level (26), which in turn affects adult health. Third, maltreatment may lead to risky behaviours (e.g. smoking (27) or alcohol use (28)) which impact on disease risk. Fourth, the influence of maltreatment on mental health problems including depression (26), could link to cardiometabolic biomarkers via increased physiologic response to stressors (29) or less directly via unhealthy behaviours (3). We examined these potential intermediaries in a general population birth cohort. Our study aims were to investigate (i) whether different forms of childhood maltreatment (neglect and abuse) were associated with adult cardiometabolic measures (obesity, BP, blood lipids and glycated haemaglobin) independent of other key early-life factors such as birthweight and SEP; and (ii) whether intermediary factors (life-course BMI gains, adult SEP, unhealthy behaviours, poor mental health) could account for associations observed.

For peer review only - http://bmjopen.bmj.com/site/about/guidelines.xhtml

Methods

The 1958 British birth cohort includes all births during one week March 1958 (n=17,638) and 920 immigrants with the same birth week recruited to 16y. Information was collected throughout childhood (7, 11, 16y) and adulthood (23, 33, 42, 45, 50, 55y). At 45y, 11,971 individuals in contact with the study were invited to a home-based clinical assessment; 9,377 (78%) participants who provided information were broadly representative of the surviving cohort (30). The 45y survey included a childhood maltreatment questionnaire, to which participants gave written informed consent. Ethical approval was given by the South-East Multi-Centre Research Ethics Committee (ref:01/1/44).

Childhood maltreatment: Information was ascertained during childhood and at 45y (**Table S1**). Neglect was based on structured questionnaires; a scale (range 0-5) was derived, separately for 7y and 11y, by summing five items on parental involvement and the child's physical appearance; a binary measure was derived as ≥2 items (7y and/or 11y). Child abuse (physical, sexual or psychological) by a parent to 16y (yes/no) was reported in adulthood (45y) in a confidential self-complete questionnaire (25) (details in **Table S1**).

Outcomes: cardiometabolic measurements were obtained at 45y by nurses using standardized protocols. Height, weight and waist circumference were measured; BMI was derived as kg/m^2. BP was measured three times while seated after five mins rest using an Omron 705CP automated digital oscillometric sphygmomanometer; means of three systolic BP (SBP) and diastolic BP (DBP) measurements were calculated. Non-fasting venous blood samples were collected and analysed for total and high density lipoprotein cholesterol (HDL-c) and triglyceride concentrations using an autoanalyzer (Olympus AU640; Tokyo) with enzymatic methods. Low density lipoprotein cholesterol (LDL-c) was calculated using Friedewald's formula (31) when triglyceride concentration<4.5mmol/L. Glycated hemoglobin (HbA_{1c}) concentrations were measured in whole citrated blood using ion-exchange HPLC. Nurses recorded information on prescribed medications: anti-hypertensive (n=429), lipid-regulating (n=166), oral glucose-lowering/antidiabetic (type 2 (T2) diabetes) (n=111); T2

For peer review only - http://bmjopen.bmj.com/site/about/guidelines.xhtml

diabetes was also identified from self-reported doctor diagnosis at 42y (n=134) excluding type 1 diabetes (n=57).

Cardiometabolic biomarkers (binary variables) were derived using established cut-offs (**Table 1**) from the literature(32;33) and our previous studies (34). These include general obesity (BMI≥30kg/m^2) and central obesity (waist circumference ≥102cm (males), ≥88cm (females)(32)), hypertension (SBP/DBP≥140/90mmHg or anti-hypertensive medication(35)), high triglycerides (>2.3mmol/L(36)), low HDL-c (≤1.0mmol/L (males), ≤1.3mmol/L (females)), and high LDL-c (>4.13mmol/L(33)). High triglycerides, low HDL-c, and high LDL-c groups also include individuals on lipid-regulating medications. Elevated (≥6%) HbA_{1c} (37), and T2 diabetes were grouped together, excluding T1 diabetes. Metabolic syndrome was defined using modified criteria of the Third Report of the National Cholesterol Education Program's Adult Treatment Panel (33) as ≥3 of: central obesity, high BP, low HDL-c, high triglycerides, and elevated HbA_{1c} (or T2 diabetes) as defined above.

Covariates: were selected a-priori from the literature based on relationships with child maltreatment and adult cardiometabolic biomarkers, including factors affecting (measurement of) outcomes, early-life factors, and potential intermediary factors. Early-life factors were birthweight for gestational age, childhood social class, household crowding and housing tenure. Birthweight for gestational age was calculated as birthweight standardized within gestational week. Social class was based on father's occupation at birth (or at 7y if missing), classified as I/II; IIINM; IIIM; IV/V (including no male head). Household overcrowding (≥1.5 persons per room) and social housing (rented from the council or housing association, versus owner occupied or privately rented) at 7y were used as measures of material (dis)advantage.

Factors affecting (measurement of) outcomes included room temperature (for BP), month of examination, time of day of blood collection, time since last meal, postal delay of blood sample (for lipids and HbA_{1c}), oral contraception (10.8%) and HRT use (7.4%) for females (for all outcomes), family history of T2 diabetes (2.4%) (for obesity and HbA_{1c}). All factors affecting measurement of the outcome were included in the analysis of metabolic syndrome.

For peer review only - http://bmjopen.bmj.com/site/about/guidelines.xhtml

Intermediary factors for the maltreatment/cardiometabolic markers relationship included childhood (7y) and adult (45y) BMI, adult SEP (social class, educational qualifications), adult lifestyles (smoking, alcohol consumption, physical activity) and mental health as indexed by depressive symptoms. Adult social class was based on the participant's occupation at 42y (or at 33y if missing). Educational qualifications attained by 33y, was categorised into: degree/higher; A-level; O-level; <O-level; no qualifications. Smoking status was reported at 42y (or at 33y if missing at 42y). Alcohol consumption frequency was reported at 42y, classified as: non-drinker; ≤once/month; ≤3 time/month; once/week; 2-3 days/week; most days. Leisure-time physical activity, ascertained at 42y from questions on how often cohort members participated in a range of activities, was categorised as: active≤3 times/month; 1 day/week; 2–3 days/week; 4–7 days/week. For depressive symptoms a 0-15 scale was derived from 15 items included in the Malaise Inventory at 42y.

Analysis

Associations of each child maltreatment with continuous and binary outcomes were examined using linear and logistic regressions respectively, for genders combined, except where associations differed (p≤0.05 for gender*maltreatment interaction) which were analysed separately. Continuous triglycerides and HbA_{1c} measures were log-transformed to correct skewness of distributions; % change in mean level approximates 100*(regression coefficient)(38). Models included corrections for effects of medication to minimise potential bias in estimates: (i) for antihypertensive medication we added a constant of 10mmHg to observed SBP and DBP (39); (ii) lipid levels were corrected assuming that lipid-lowering drugs reduce total cholesterol by 20%, LDL-c by 35%, triglycerides by 15% and increase HDL-c by 5%, based on average efficacy of statins, the most frequently prescribed lipid-lowering drug in this study (40); (iii) for antidiabetic medication HbA_{1c} levels were corrected assuming that medication reduced HbA_{1c} levels by 1% in absolute terms (41).

Baseline models included gender and factors affecting measurements (model 1). Models were then adjusted for early-life factors (and, for HbA_{1c}, family history of T2 diabetes) (model 2). For

For peer review only - http://bmjopen.bmj.com/site/about/guidelines.xhtml

associations that remained in model 2 ($p<0.05$, or borderline for sexual abuse due to low prevalence), we further adjusted for potential intermediary factors. To test the separate and combined intermediary role of these factors, we examined models that further adjusted for: (1) BMI at 7 and 45y, (2) SEP (social class, educational qualifications), (3) lifestyle factors (smoking, alcohol consumption, physical activity), (4) depressive symptoms, and (5) all factors simultaneously (models 3-7). However, conventional regression models with adjustment for potential mediators could possibly induce bias e.g., via exposure-mediator interactions (42). Hence, we conducted selected checks to ensure that associations were unaffected by such biases: (i) tests of maltreatment-intermediary factor interaction(s) which were mostly non-significant; (ii) adjustments to test mediator-outcome confounding: we examined effects on parameter estimates of additional adjustment for adult SEP which is associated with both intermediary factors (e.g. lifestyles) and cardiometabolic outcomes (40); effects of such adjustments were negligible. As an additional check we conducted mediation analysis using Inverse Odds Ratio Weighting method (43). General patterns of mediation effects were consistent with those obtained from regression models 3-7 and conclusions remained unchanged. Hence we present results using conventional regression with adjustment for mediators for simplicity of interpretation.

In addition to analyses of specific types, we examined cumulative burden of maltreatment, as in previous research (4); we summed the number of types of maltreatment and, using linear regression, examined associations with cardiometabolic measures, with and without adjustment for early-life factors.

Among participants with cardiometabolic markers (n=7391-9297 depending on outcome), 93% had information on neglect and nearly all on abuse. To maximise available information, we applied multiple imputation to missing data on covariates and neglect to the sample alive to 45y (N=17313). Imputation models included analysis variables (maltreatment, cardiometabolic markers, covariates) and predictors of non-response (30) (gender, ethnicity, class at birth, 7y reading ability). We created 25 imputed datasets assuming missing at random given other variables in the imputation models.

Analysis samples were restricted to individuals with observed cardiometabolic markers. Parameters from imputed datasets were combined to obtain overall estimates using Rubin's rules (44).

Results

Approximately 12% of participants reported any childhood abuse: 6% (physical), 1.6% (sexual), and 10% (psychological) abuse, 17.4% were classified as neglected; most were not maltreated, 20.4% had one and 6.1% had ≥2 types (**Table 1**).

Childhood neglect was associated with several cardiometabolic biomarkers after adjusting for factors affecting measurement and early-life covariates: BMI and waist circumference were higher by 0.53 (95% CI:0.23,0.83) kg/m^2 and 1.23(0.51,1.96) cm respectively, ORs were higher for both general (1.16(1.02,1.32)) and central 1.15(1.02,1.30) obesity; triglycerides were raised by 3.9(0.3,7.5)% and HbA_{1c} by 1.2(0.4,2.0)%, and, among females, HDL-c was lower by 0.05(0.01,0.08) mmol/L (**Table 2**). When considering potential intermediary factors, associations reduced (mostly disappeared) after further adjusting for child-to-adult BMI, adult SEP and lifestyles (mainly smoking and alcohol consumption rather than physical activity) (**Tables 3** and **S2**).

Physical abuse was associated with higher BMI (by 0.72(0.28,1.16) kg/m^2), waist circumference (by 1.29(0.23,2.35)cm) and obesity (OR=1.36(1.13,1.64) for general obesity; 1.38(1.16,1.65) for central obesity); increased risk of high LDL-c (OR=1.25(1.00,1.56)), raised HbA_{1c} in males (by 2.5(0.7,4.3)%), and lower HDL-c in females (by 0.06(0.01,0.12)mmol/L) (**Table 2**). In relation to potential intermediary factors, associations largely attenuated when adjusting for adult lifestyles (notably smoking), and child-to-adult BMI (for HDL-c in females) except for BMI, waist and obesity which were unaffected (**Tables 3** and **S2**). *Sexual abuse* associations with biomarkers were similar or greater in magnitude than other maltreatments, e.g. physical abuse, but CIs were wide due to few cases (**Table 2**): e.g., OR of high LDL-c was 1.41(0.89,2.23) after adjustment for early-life factors, attenuating to 1.26(0.79,2.00) when adjusting for lifestyles, predominantly smoking (**Tables 3** and **S2**). *Psychological abuse* was associated with elevated risk of high triglyceride levels

For peer review only - http://bmjopen.bmj.com/site/about/guidelines.xhtml

(OR=1.21(1.02,1.44)) and low HDL-c (by 0.04(0.01,0.07)mmol/L) (**Table 2**). When considering potential intermediary factors, associations disappeared when adjusting for lifestyles (mostly smoking) and mental health (**Tables 3** and **S2**).

Childhood maltreatments were not associated with raised BP or risk of metabolic syndrome, but since prevalence is low for sexual abuse we cannot rule out elevated risks for either metabolic syndrome or T2 diabetes (**Table 2**). Intriguingly, sexual abuse was associated with a reduced risk for hypertension (OR=0.43 (0.24, 0.74)); additional analysis for pulse pressure showed similar findings (results not presented). Whereas for other maltreatments, the null findings for BP, hypertension, metabolic syndrome or T2 diabetes are less likely to be due to lack of statistical power. Finally, few associations were found between number of types of maltreatment and cardiometabolic outcomes after adjusting for early-life factors; exceptions included BMI and elevated triglyceride levels, the latter reflecting weak and mostly non-significant associations across all maltreatment types (**Table 4** and **S3**).

Discussion

Main findings include, first, all forms of child maltreatment were associated with at least one adverse cardiometabolic outcome (adiposity, blood lipids and/or HbA_{1c}) in mid-adulthood, although there was no evidence of adverse effects on BP. Importantly, associations were independent of birthweight and early-life SEP shown elsewhere to be associated with biomarkers for cardiometabolic disease (1;2). Associations were consistent for neglect and physical abuse in relation to adiposity, whilst for other maltreatments and outcomes associations were few, effects were modest, and evidence was weak for accumulative associations for number of types of maltreatment. Second, in relation to potential intermediary factors for the child maltreatment-cardiometabolic associations observed, these mostly disappeared after adjusting for adult lifestyles, suggesting that lifestyles may play a key mediating role, whilst adult SEP was important for the association of neglect but not physical abuse with adiposity.

Methodological considerations

For peer review only - http://bmjopen.bmj.com/site/about/guidelines.xhtml

Study strengths include a population-based cohort followed-up over five decades, with a range of adult cardiometabolic measures, prospectively ascertained childhood covariates including multiple indicators of family SEP, and potential intermediary factors. Measurement of childhood neglect was recorded prospectively from multiple sources; items relate to some dimensions (failure to meet a child's basic physical, emotional and education needs) but not all aspects (e.g. inadequate nutrition or shelter) of neglect (45). As individual items may not imply neglectful behaviour, we used a score of ≥two items. Such measures are associated with delayed childhood height growth (46), cognitive ability and poorer qualifications (47) in this population, as expected from the wider literature using differing study designs and thereby supporting construct validity for our neglect measures. Childhood abuse was self-reported at 45y. All ascertainment methods for child maltreatment have limitations (45). Parental report may be influenced by socially desirable responding and miss cases due to under-reporting, likewise only a small proportion of cases are identified by agencies. Whilst adult report of abuse may be affected by recall bias or current emotional state (48), retrospective report provides an accepted method in population studies (45); in the 1958 cohort it was blind to knowledge of issues to be investigated and data show expected associations with prospectively measured family dysfunction (49) and mental health (47), suggesting good construct validity. Moreover, it has been suggested that retrospective reports show less biased associations with objective adult outcomes (such as the risk factors for cardiometabolic disease examined here) than self-reported outcomes (50). Some cardiometabolic measures were collected from non-fasted blood. Such measures may be inappropriate for clinical purposes (triglycerides levels tend to be lower in non-fasted samples (51) and vary by fasting duration and time of day (52)) but are often adequate for population studies given that fasting and non-fasting levels (e.g. of triglycerides) are positively correlated (53) and because non-fasting levels have been found to be a significant risk factor for cardiovascular disease (54;55). Moreover, misclassification of lipid or triglyceride levels by our exposures is unlikely, and we adjusted for time of last meal and blood collection in analyses for lipids and HbA_{1c}. Sample attrition had occurred over time. We applied multiple imputation for missing covariates. Finally, study power for sexual abuse may be inadequate for detecting associations due to low prevalence. Regarding methodological approach to assess potential intermediary factors for maltreatment-cardiometabolic associations, we

For peer review only - http://bmjopen.bmj.com/site/about/guidelines.xhtml

used conventional regression and confirmed the robustness of our conclusions using additional checks (including Inverse Odds Ratio Weighting method (43)).

Interpretation and comparison with other studies

Our main finding of few modest associations between types of child maltreatment and biomarkers for cardiometabolic disease adds to the literature in several important respects. First, the adverse associations found for adult adiposity, blood lipids and HbA_{1c} but not BP, suggests the primary biological pathways through which some maltreatments could have deleterious effects on later cardiometabolic morbidity and mortality. These findings agree with others in their emphasis on adiposity (14) and lipids/glucose (17-19), and whilst several studies report associations with BP (10;12;16), not all do (11;15). One possibility is that some BP findings reported to date are conflated by lack of control for other early-life factors, a short-coming that we have been able to address. Moreover, others suggest, as we do, that associations are small to modest (6). Similar to our findings, the CARDIA study reported that 'risky' family environment (cold/unaffectionate family interactions, conflict, aggression, neglect, low nurturance) was associated with HDL-c level in women, but not with BP (6). Specific types of maltreatment in our study, namely neglect and physical abuse, appeared to be particularly relevant to cardiometabolic outcomes, although as mentioned above associations for sexual abuse were comparable in magnitude. Comparison with existing research is hindered by the dearth of studies that distinguish neglect from other maltreatments, but our associations of neglect and physical abuse with increased HbA_{1c} (potentially T2 diabetes) agree with evidence to date (17-19) and results for physical/sexual abuse are consistent with an increased CVD risk for women (7). Unlike others (4), we found little evidence of associations for number of types of maltreatment, a null finding that supports the suggestion of there being a few specific associations rather than cumulation across several types of maltreatment. Our findings contrast with previous research in other respects, e.g., the lack of association for non-sexual abuse and metabolic syndrome (despite associations for key lipid and glucose components) contrasts with reported increased risk for physical abuse (15;20). Such discrepancies may arise from our null findings for BP and other considerations such as differences in design (e.g. self-reported hypertension (10;12)) or in age (12;16). Associations for maltreatment may

For peer review only - http://bmjopen.bmj.com/site/about/guidelines.xhtml

vary by age, emerging later in life, as suggested by studies showing rapid gains in BP (13) and in BMI from early adulthood (23).

For our second main finding, results suggest that adult lifestyle was a key proximal intermediary factor for several maltreatment–biomarker associations, which concurs with some (4;6;7;56), though not all (10;18) previous studies. Our interpretation of this finding is that it suggests a 'causal chain' whereby child neglect and abuse are associated with higher rates of smoking and obesity (10;27;57) that, in turn, affect cardiometabolic markers such as lipids and HbA_{1c}. Concordant with our results, the US Nurses Health Study found that adult lifestyle and medical risk factors accounted for much of the association between child abuse and adult CVD (7). In relation to other potential explanations, we would expect adult SEP to be an important factor, as reported elsewhere (58), especially for neglect rather than non-sexual abuse given its association with lower adult SEP in this cohort (25). Our results confirm this expectation, as several associations for neglect (less so for abuse) attenuated after allowing for adult SEP. Of the other hypothesised intermediary factors examined, child-to-adult BMI trajectory appeared to contribute to associations for neglect and physical abuse with adult lipid and HbA_{1c} levels. This agrees with the US Nurses Health Study in which adult BMI was found to account for most of the associations between physical/sexual abuse and adult T2 diabetes (18). Lastly for mental health, the suggestion in our study that this was an important pathway for psychological abuse is unsurprising. We might have expected it to impact other maltreatment- biomarker associations but found only negligible effects, although psychological processes other than depressive symptomatology could contribute.

Conclusions and implications

Childhood maltreatments, particularly neglect and physical abuse, were associated with greater adiposity and poorer lipid and HbA_{1c} profiles in mid-adulthood, suggesting further long-term health consequences for these groups. Although associations were only modest, importantly, they were independent of main early-life factors (birthweight, SEP) linked previously to these outcomes. Study findings suggest that adult lifestyles and child-to-adult BMI may be key intermediaries for many

For peer review only - http://bmjopen.bmj.com/site/about/guidelines.xhtml

associations, with some evidence for additional factors, such as adult SEP. Whether these intermediary risk factors among child maltreatment groups would benefit from standard interventions (e.g. for smoking cessation) in adulthood remains to be answered. Such interventions may be ineffective, if for example, the propensity to smoke or to gain BMI is biologically embedded from an early age and is difficult to alter thereafter. Further work is needed on the role of life-course intermediary factors to better understand the pathways and mechanisms underlying maltreatments-cardiometabolic disease associations, including consideration of differing as well as common pathways for specific maltreatments. These efforts are essential to effective strategies to reduce or prevent adult health consequences of child maltreatment.

Conflict of interest: None to declare.

Abbreviations:

Cardiovascular disease (CVD)

Adverse childhood experiences (ACE)

Odds ratio (OR)

Blood pressure (BP)

Socio-economic position (SEP)

Body mass index (BMI)

High density lipoprotein cholesterol (HDL-c)

Low density lipoprotein cholesterol (LDL-c)

Glycated haemoglobin (HbA_{1c})

Ethics approval and consent to participate: The 45y survey included a childhood maltreatment questionnaire, to which participants gave written informed consent. Ethical approval was given by the South-East Multi-Centre Research Ethics Committee (ref:01/1/44).

Consent for publication: Not applicable.

Availability of data and material

For peer review only - http://bmjopen.bmj.com/site/about/guidelines.xhtml

NCDS data used in this research are available from the UK Data Archive (http://www.data-archive.ac.uk); applications for access to any data that forms part of the NCDS Biomedical Resource will require special license and should be submitted to clsfeedback@ioe.ac.uk.

Funding sources

This work was funded by the Department of Health Policy Research Programme through the Public Health Research Consortium (PHRC) and supported by the National Institute for Health Research Biomedical Research Centre at Great Ormond Street Hospital for Children NHS Foundation Trust and University College London. The views expressed in the publication are those of the authors and not necessarily those of the Department of Health. Information about the wider programme of the PHRC is available from http://phrc.lshtm.ac.uk. Data collection for participants at age 45 was funded by the Medical Research Council, grant G0000934. The authors are grateful to the Centre for Longitudinal Studies (CLS), University College London Institute of Education for the use of these data and to the UK Data Service for making them available. However, neither CLS nor the UK Data Service bears any responsibility for the analysis or interpretation of these data.

Author contributions

LL conceptualised the study, carried out the analyses, drafted the manuscript, revised it critically for important intellectual content and approved the final version to be published.

CP conceptualised the study, drafted the manuscript, revised it critically for important intellectual content and approved the final version to be published.

SMPP revised the paper critically for important intellectual content and approved the final version to be published.

For peer review only - http://bmjopen.bmj.com/site/about/guidelines.xhtml

Table legends:

Table 1: Childhood maltreatment and adult cardiometabolic biomarkers (mean (SD) or %)

Table 2: Associations (mean difference or OR) between childhood maltreatment and adult cardiometabolic markers unadjusted and adjusted for early-life factors

Table 3: Associations (mean difference or OR) for childhood maltreatment and adult cardiometabolic markers, adjusted for intermediary factors, separately and combined

Table 4: Associations (mean difference or OR) between number of maltreatments and cardiometabolic markers at 45y

For peer review only

For peer review only - http://bmjopen.bmj.com/site/about/guidelines.xhtml

Reference List

(1) Huxley R, Owen CG, Whincup PH, Cook DG, Colman S, Collins R. Birth weight and subsequent cholesterol levels: exploration of the "fetal origins" hypothesis. JAMA 2004 Dec 8;292(22):2755-64.

(2) Power C, Kuh D, Morton S. From developmental origins of adult disease to life course research on adult disease and aging: insights from birth cohort studies. Annu Rev Public Health 2013;34:7-28.

(3) Norman RE, Byambaa M, De R, Butchart A, Scott J, Vos T. The long-term health consequences of child physical abuse, emotional abuse, and neglect: a systematic review and meta-analysis. PLoS Med 2012;9(11):e1001349.

(4) Dong M, Giles WH, Felitti VJ, Dube SR, Williams JE, Chapman DP, et al. Insights into causal pathways for ischemic heart disease: adverse childhood experiences study. Circulation 2004 Sep 28;110(13):1761-6.

(5) Gilbert LK, Breiding MJ, Merrick MT, Thompson WW, Ford DC, Dhingra SS, et al. Childhood adversity and adult chronic disease: an update from ten states and the District of Columbia, 2010. Am J Prev Med 2015 Mar;48(3):345-9.

(6) Loucks EB, Almeida ND, Taylor SE, Matthews KA. Childhood family psychosocial environment and coronary heart disease risk. Psychosom Med 2011 Sep;73(7):563-71.

(7) Rich-Edwards JW, Mason S, Rexrode K, Spiegelman D, Hibert E, Kawachi I, et al. Physical and sexual abuse in childhood as predictors of early-onset cardiovascular events in women. Circulation 2012 Aug 21;126(8):920-7.

(8) Chen E, Turiano NA, Mroczek DK, Miller GE. Association of Reports of Childhood Abuse and All-Cause Mortality Rates in Women. JAMA Psychiatry 2016 Sep 1;73(9):920-7.

(9) Mehta LS, Beckie TM, DeVon HA, Grines CL, Krumholz HM, Johnson MN, et al. Acute Myocardial Infarction in Women: A Scientific Statement From the American Heart Association. Circulation 2016 Mar 1;133(9):916-47.

(10) Riley EH, Wright RJ, Jun HJ, Hibert EN, Rich-Edwards JW. Hypertension in adult survivors of child abuse: observations from the Nurses' Health Study II. J Epidemiol Community Health 2010 May;64(5):413-8.

(11) Gooding HC, Milliren C, McLaughlin KA, Richmond TK, Katz-Wise SL, Rich-Edwards J, et al. Child maltreatment and blood pressure in young adulthood. Child Abuse Negl 2014 Nov;38(11):1747-54.

(12) Stein DJ, Scott K, Haro Abad JM, Aguilar-Gaxiola S, Alonso J, Angermeyer M, et al. Early childhood adversity and later hypertension: data from the World Mental Health Survey. Ann Clin Psychiatry 2010 Feb;22(1):19-28.

(13) Su S, Wang X, Pollock JS, Treiber FA, Xu X, Snieder H, et al. Adverse childhood experiences and blood pressure trajectories from childhood to young adulthood: the Georgia stress and Heart study. Circulation 2015 May 12;131(19):1674-81.

(14) Noll JG, Zeller MH, Trickett PK, Putnam FW. Obesity risk for female victims of childhood sexual abuse: a prospective study. Pediatrics 2007 Jul;120(1):e61-e67.

(15) Midei AJ, Matthews KA, Chang YF, Bromberger JT. Childhood physical abuse is associated with incident metabolic syndrome in mid-life women. Health Psychol 2013 Feb;32(2):121-7.

(16) McIntyre RS, Soczynska JK, Liauw SS, Woldeyohannes HO, Brietzke E, Nathanson J, et al. The association between childhood adversity and components of metabolic syndrome in

For peer review only - http://bmjopen.bmj.com/site/about/guidelines.xhtml

adults with mood disorders: results from the international mood disorders collaborative project. Int J Psychiatry Med 2012;43(2):165-77.

(17) Afifi TO, Mota N, MacMillan HL, Sareen J. Harsh physical punishment in childhood and adult physical health. Pediatrics 2013 Aug;132(2):e333-e340.

(18) Rich-Edwards JW, Spiegelman D, Lividoti Hibert EN, Jun HJ, Todd TJ, Kawachi I, et al. Abuse in childhood and adolescence as a predictor of type 2 diabetes in adult women. Am J Prev Med 2010 Dec;39(6):529-36.

(19) Scott KM, Von KM, Angermeyer MC, Benjet C, Bruffaerts R, de GG, et al. Association of childhood adversities and early-onset mental disorders with adult-onset chronic physical conditions. Arch Gen Psychiatry 2011 Aug;68(8):838-44.

(20) Lee C, Tsenkova V, Carr D. Childhood trauma and metabolic syndrome in men and women. Soc Sci Med 2014 Mar;105:122-30.

(21) Lissau I, Sorensen TI. Parental neglect during childhood and increased risk of obesity in young adulthood. Lancet 1994 Feb 5;343(8893):324-7.

(22) Batten SV, Aslan M, Maciejewski PK, Mazure CM. Childhood maltreatment as a risk factor for adult cardiovascular disease and depression. J Clin Psychiatry 2004 Feb;65(2):249-54.

(23) Power C, Pinto Pereira SM, Li L. Childhood maltreatment and BMI trajectories to mid-adult life: follow-up to age 50 y in a British birth cohort. PLoS One 2015;10(3):e0119985.

(24) Vamosi M, Heitmann BL, Kyvik KO. The relation between an adverse psychological and social environment in childhood and the development of adult obesity: a systematic literature review. Obes Rev 2010 Mar;11(3):177-84.

(25) Pinto Pereira SM, Li L, Power C. Child Maltreatment and Adult Living Standards at 50 Years. Pediatrics 2017 Jan;139(1).

(26) Geoffroy MC, Pinto PS, Li L, Power C. Child Neglect and Maltreatment and Childhood-to-Adulthood Cognition and Mental Health in a Prospective Birth Cohort. J Am Acad Child Adolesc Psychiatry 2016 Jan;55(1):33-40.

(27) Jun HJ, Rich-Edwards JW, Boynton-Jarrett R, Austin SB, Frazier AL, Wright RJ. Child abuse and smoking among young women: the importance of severity, accumulation, and timing. J Adolesc Health 2008 Jul;43(1):55-63.

(28) Widom CS, White HR, Czaja SJ, Marmorstein NR. Long-term effects of child abuse and neglect on alcohol use and excessive drinking in middle adulthood. J Stud Alcohol Drugs 2007 May;68(3):317-26.

(29) Miller GE, Chen E, Parker KJ. Psychological stress in childhood and susceptibility to the chronic diseases of aging: moving toward a model of behavioral and biological mechanisms. Psychol Bull 2011 Nov;137(6):959-97.

(30) Atherton K, Fuller E, Shepherd P, Strachan DP, Power C. Loss and representativeness in a biomedical survey at age 45 years: 1958 British birth cohort. J Epidemiol Community Health 2008 Mar;62(3):216-23.

(31) Friedewald WT, Levy RI, Fredrickson DS. Estimation of the concentration of low-density lipoprotein cholesterol in plasma, without use of the preparative ultracentrifuge. Clin Chem 1972 Jun;18(6):499-502.

(32) Clinical Guidelines on the Identification, Evaluation, and Treatment of Overweight and Obesity in Adults--The Evidence Report. National Institutes of Health. Obes Res 1998 Sep;6 Suppl 2:51S-209S.

For peer review only

For peer review only - http://bmjopen.bmj.com/site/about/guidelines.xhtml

(33) Executive Summary of The Third Report of The National Cholesterol Education Program (NCEP) Expert Panel on Detection, Evaluation, And Treatment of High Blood Cholesterol In Adults (Adult Treatment Panel III). JAMA 2001 May 16;285(19):2486-97.

(34) Li L, Pinot de MA, Power C. Predicting cardiovascular disease risk factors in midadulthood from childhood body mass index: utility of different cutoffs for childhood body mass index. Am J Clin Nutr 2011 Jun;93(6):1204-11.

(35) 1999 World Health Organization-International Society of Hypertension Guidelines for the Management of Hypertension. Guidelines Subcommittee. J Hypertens 1999 Feb;17(2):151-83.

(36) Miller M, Stone NJ, Ballantyne C, Bittner V, Criqui MH, Ginsberg HN, et al. Triglycerides and cardiovascular disease: a scientific statement from the American Heart Association. Circulation 2011 May 24;123(20):2292-333.

(37) Thomas C, Hypponen E, Power C. Prenatal exposures and glucose metabolism in adulthood: are effects mediated through birth weight and adiposity? Diabetes Care 2007 Apr;30(4):918-24.

(38) Cole TJ. Sympercents: symmetric percentage differences on the 100 log(e) scale simplify the presentation of log transformed data. Stat Med 2000 Nov 30;19(22):3109-25.

(39) Tobin MD, Sheehan NA, Scurrah KJ, Burton PR. Adjusting for treatment effects in studies of quantitative traits: antihypertensive therapy and systolic blood pressure. Stat Med 2005 Oct 15;24(19):2911-35.

(40) Ki M, Pouliou T, Li L, Power C. Physical (in)activity over 20 y in adulthood: associations with adult lipid levels in the 1958 British birth cohort. Atherosclerosis 2011 Nov;219(1):361-7.

(41) Bennett WL, Maruthur NM, Singh S, Segal JB, Wilson LM, Chatterjee R, et al. Comparative effectiveness and safety of medications for type 2 diabetes: an update including new drugs and 2-drug combinations. Ann Intern Med 2011 May 3;154(9):602-13.

(42) Richiardi L, Bellocco R, Zugna D. Mediation analysis in epidemiology: methods, interpretation and bias. Int J Epidemiol 2013 Oct;42(5):1511-9.

(43) Nguyen QC, Osypuk TL, Schmidt NM, Glymour MM, Tchetgen Tchetgen EJ. Practical guidance for conducting mediation analysis with multiple mediators using inverse odds ratio weighting. Am J Epidemiol 2015 Mar 1;181(5):349-56.

(44) Schafer JL. Multiple imputation: a primer. Stat Methods Med Res 1999 Mar;8(1):3-15.

(45) Gilbert R, Widom CS, Browne K, Fergusson D, Webb E, Janson S. Burden and consequences of child maltreatment in high-income countries. Lancet 2009 Jan 3;373(9657):68-81.

(46) Denholm R, Power C, Li L. Adverse childhood experiences and child-to-adult height trajectories in the 1958 British birth cohort. Int J Epidemiol 2013 Oct;42(5):1399-409.

(47) Geoffroy MC, Pinto PS, Li L, Power C. Child Neglect and Maltreatment and Childhood-to-Adulthood Cognition and Mental Health in a Prospective Birth Cohort. J Am Acad Child Adolesc Psychiatry 2016 Jan;55(1):33-40.

(48) Fergusson DM, Horwood LJ, Woodward LJ. The stability of child abuse reports: a longitudinal study of the reporting behaviour of young adults. Psychol Med 2000 May;30(3):529-44.

(49) Denholm R, Power C, Thomas C, Li L. Child maltreatment and household dysfunction in a British birth cohort. Child Abuse Review 2013;(In press; DOI: 10.1002/car.2235).

(50) Reuben A, Moffitt TE, Caspi A, Belsky DW, Harrington H, Schroeder F, et al. Lest we forget: comparing retrospective and prospective assessments of adverse childhood experiences in the prediction of adult health. J Child Psychol Psychiatry 2016 Oct;57(10):1103-12.

For peer review only - http://bmjopen.bmj.com/site/about/guidelines.xhtml

(51) Wilder LB, Bachorik PS, Finney CA, Moy TF, Becker DM. The effect of fasting status on the determination of low-density and high-density lipoprotein cholesterol. Am J Med 1995 Oct;99(4):374-7.

(52) Emberson JR, Whincup PH, Walker M, Thomas M, Alberti KG. Biochemical measures in a population-based study: effect of fasting duration and time of day. Ann Clin Biochem 2002 Sep;39(Pt 5):493-501.

(53) Zweers A, Yaron E, Groen JJ. A study of fasting and postprandial serum triglycerides in connection with epidemiological surveys. Clin Chim Acta 1968 Feb;19(2):267-75.

(54) Nordestgaard BG, Benn M, Schnohr P, Tybjaerg-Hansen A. [Non-fasting triglycerides and risk of for myocardial infarction and death among women and men]. Ugeskr Laeger 2007 Nov 5;169(45):3865-8.

(55) Bansal S, Buring JE, Rifai N, Mora S, Sacks FM, Ridker PM. Fasting compared with nonfasting triglycerides and risk of cardiovascular events in women. JAMA 2007 Jul 18;298(3):309-16.

(56) Kendall-Tackett K. The health effects of childhood abuse: four pathways by which abuse can influence health. Child Abuse Negl 2002 Jun;26(6-7):715-29.

(57) Power C, Pinto Pereira SM, Li L. Childhood maltreatment and BMI trajectories to mid-adult life: follow-up to age 50 y in a British birth cohort. PLoS One 2015;10(3):e0119985.

(58) Friedman EM, Karlamangla AS, Gruenewald TL, Koretz B, Seeman TE. Early life adversity and adult biological risk profiles. Psychosom Med 2015 Feb;77(2):176-85.

For peer review only

For peer review only - http://bmjopen.bmj.com/site/about/guidelines.xhtml

Table 1: Childhood maltreatment and adult cardiometabolic biomarkers (mean(SD) or %)

	N	Males	Females
**Childhood maltreatment**			
Neglect†	8734	18.9%	16.0%
Abuse			
Physical	9309	6.0%	6.2%
Sexual	9309	0.5%	2.7%
Psychological	9310	8.3%	11.6%
**Number of types**			
0	6371	73.4%	73.5%
1	1771	21.3%	19.5%
2	384	4.1%	4.7%
≥3	150	1.2%	2.3%
**Cardiometabolic markers(45y)‡**			
BMI (kg/m^2)	9348	27.8(4.4)	27.0(5.6)
General obesity(≥30kg/m^2)		25.4%	23.7%
Waist circumference(cm)	9291	98.5(11.2)	85.6(12.9)
Central obesity(≥102/88cm)$		32.7%	36.9%
Blood pressure(mmHg)			
SBP	9297	133.3(15.4)	120.8(16.0)
DBP	9297	82.5(10.8)	76.1(10.8)
High SBP/DBP(≥140/90)	9297	34.6%	16.3%
Blood lipids(mmol/L)			
Total cholesterol	7824	6.10(1.17)	5.71(1.02)
HDL-cholesterol	7808	1.43(0.34)	1.69(0.41)
Low(≤1.0/1.3) HDL-c$	7847	11.8%	20.3%
LDL-cholesterol	7391	3.60(0.96)	3.30(0.89)
High(>4.13) LDL-c	7451	27.1%	15.8%
Triglycerides	7799	2.52(1.83)	1.60(1.14)
High(>2.3) triglycerides	7837	42.7%	16.7%
HbA_{1c}(%)	7923	5.34(0.82)	5.20(0.68)
High(>6%) HbA_{1c}	7964	6.1%	4.2%
Metabolic syndrome	7640	15.1%	10.2%

†≥2 indicators (7 and/or 11y); sample for individuals with cardiometabolic marker(s)
‡all cardiometabolic markers (continuous measures) were adjusted for medication. For binary outcomes (hypertension, dyslipidemia, or T2 diabetes), those on medication were in risk groups
$cut-offs for males/females.

For peer review only - http://bmjopen.bmj.com/site/about/guidelines.xhtml

Table 2: Associations (mean difference or OR) between childhood maltreatment and adult cardiometabolic markers unadjusted and adjusted for early-life factors

	Neglect		Physical abuse		Sexual abuse		Psychological abuse	
	Model 1	Model 2	Model 1	Model 2	Model 1	Model 2	Model 1	Model 2
**Mean difference(95%CI)‡**								
BMI(kg/m^2)	**0.87(0.59,1.15)**	**0.53(0.23,0.83)**	**0.83(0.39,1.27)**	**0.72(0.28,1.16)**	0.34(-0.50,1.18)	0.14(-0.70,0.97)	**0.36(0.01,0.71)**	0.28(-0.07,0.63)
Waist circumference(cm)	**1.88(1.19,2.57)**	**1.23(0.51,1.96)**	**1.44(0.38,2.50)**	**1.29(0.23,2.35)**	0.15(-1.89,2.19)	-0.16(-2.19,1.88)	**0.89(0.05,1.73)**	0.74(-0.10,1.58)
**Blood pressure**								
SBP(mmHg)	**1.40(0.51,2.29)**	0.80(-0.14,1.73)	-0.46(-1.83,0.91)	-0.83(-2.20,0.54)	-2.90(-5.56,-0.24)[#]	-3.50(-6.16,-0.84)[#]	-0.28(-1.38,0.81)	-0.45(-1.54,0.65)
DBP(mmHg)	**0.99(0.38,1.59)**	0.58(-0.06, 1.22)	0.17(-0.78,1.12)	-0.08(-1.03,0.87)	-2.02(-3.84,-0.20)[#]	-2.45(-4.27,-0.63)[#]	0.54(-0.21,1.30)	0.43(-0.32,1.19)
**Blood lipids**								
Total cholesterol(mmol/L)	0.06(-0.01,0.13)	0.04(-0.030.12)	0.08(-0.03,0.18)	0.07(-0.04,0.17)	0.16(-0.06,0.38)	0.14(-0.08,0.36)	-0.02(-0.10,0.07)	-0.02(-0.11,0.07)
HDL-c(mmol/L)					-0.07(-0.14,0.01)	-0.05(-0.12,0.02)	**-0.05(-0.07,-0.02)**	**-0.04(-0.07,-0.01)**
males	**-0.03(-0.06,0)**	-0.01(-0.04,0.02)	0.0(-0.05,0.05)	0.0(-0.05,0.05)				
females	**-0.08(-0.12,-0.05)**	**-0.05(-0.08,-0.01)**	**-0.08(-0.14,-0.02)**	**-0.06(-0.12,-0.01)**				
LDL-c	**0.06(0.01,0.12)**	0.05(-0.02,0.11)	0.07(-0.02,0.17)	0.06(-0.03,0.16)	**0.19(0,0.38)**	0.17(-0.02,0.36)	0.01 (-0.07,0.08)	0(-0.08,0.08)
Triglycerides†%	**7.7(4.3,11.1)**	**3.9(0.3,7.5)**	5.5(-0.1,1.1)	3.6(-2.0,9.2)	4.5(-6.5,15.5)	1.7(-9.1,12.5)	2.6(-1.8,7.0)	1.7(-2.7,6.1)
HbA_{1c}†%	**1.7(0.9,2.5)**	**1.2(0.4,2.0)**			**2.7(0.3,5.1)**	**2.4(0.0,4.8)**	0.6(-0.4,1.6)	0.5(-0.5,2.0)
males			**2.8(1.0,4.6)**	**2.5(0.7,4.3)**				
females			0.8(-0.8,2.4)	0.5(-1.1,2.1)				
**OR(95%CI) for elevated levels$**								
General obesity	**1.32(1.17,1.49)**	**1.16(1.02,1.32)**	**1.42(1.18,1.71)**	**1.36(1.13,1.64)**	1.20(0.84,1.73)	1.12(0.77,1.61)	**1.17(1.00,1.36)**	1.13(0.97,1.32)
Central obesity	**1.27(1.13,1.43)**	**1.15(1.02,1.30)**	**1.41(1.19,1.68)**	**1.38(1.16,1.65)**	1.01(0.72,1.42)	0.96(0.68,1.36)	1.13(0.98,1.30)	1.11(0.96,1.27)
Hypertension	**1.21(1.07,1.37)**	1.12(0.98,1.27)	1.00(0.81,1.22)	0.95(0.77,1.17)	0.46(0.26,0.80)	0.43(0.24,0.74)	1.02(0.86,1.20)	1.00(0.85,1.18)
HDL-c	**1.36(1.16,1.59)**	1.15(0.98,1.36)	**1.29(1.01,1.65)**	1.20(0.94,1.54)	1.43(0.93,2.21)	1.29(0.83,2.01)	1.12(0.91,1.37)	1.08(0.88,1.32)
LDL-c	1.09(0.94,1.25)	1.04(0.89,1.21)	**1.27(1.02,1.59)**	**1.25(1.00,1.56)**	1.46(0.92,2.31)	1.41(0.89,2.23)	1.00(0.83,1.21)	0.99(0.82,1.19)
Triglycerides	**1.19(1.04,1.36)**	1.08(0.93,1.24)	**1.26(1.02,1.57)**	1.20(0.97,1.49)	1.30(0.83,2.03)	1.20(0.77,1.88)	**1.23(1.04,1.47)**	**1.21(1.02,1.44)**
HbA_{1c}	**1.43(1.11,1.84)**	1.24(0.95,1.61)	1.38(0.91,2.11)	1.27(0.83,1.94)	1.53(0.68,3.45)	1.36(0.60,3.09)	1.20(0.84,1.73)	1.16(0.81,1.67)
Metabolic syndrome	1.22(0.99,1.50)	1.08(0.89,1.32)	1.20(0.86,1.69)	1.14(0.81,1.60)	1.48(0.48,4.53)	1.37(0.45,4.20)	1.18(0.93,1.51)	1.16(0.91,1.47)

NB: genders combined except when gender*maltreatment interaction $p \leq 0.05$.
Model 1 includes gender and factors affecting measurement (for BP: room temperature; lipids and HBA1c: examination month, time of blood collection, time since last meal, postal delay of blood sample; for females: oral contraception and HRT). For metabolic syndrome: all factors included.
Model 2: model 1 factors, family history of diabetes (for HbA1c), early-life factors (birthweight-for-gestational age, social class, housing tenure, crowding at 7y)
†log-transformed and converted to % [NB: for HbA1c and triglycerides parameters are % of units (%)]
‡all cardiometabolic markers (continuous measures) were adjusted for medication. For binary outcomes (hypertension, dyslipidemia, or T2 diabetes), those on medication were in risk groups
$details of risk groups in **Table 1**
#included interaction term for gender (not separately by gender because of small Ns).

For peer review only - http://bmjopen.bmj.com/site/about/guidelines.xhtml

Table 3: Associations (mean difference or OR) for childhood maltreatment and adult cardiometabolic markers, adjusted for intermediary factors, separately and combined

	Model 2	Model 3 (+BMI7&45)	Model 4 (+adult SEP)	Model 5 (+lifestyle factors)	Model 6 (+depressive symptoms)	Model 7 (+all factors)
**Mean difference(95%CI)‡**						
**Neglect**						
BMI(kg/m^2)	**0.53(0.23,0.83)**		**0.30(0.0,0.61)**	**0.50(0.20,0.80)**	**0.50(0.20,0.80)**	**0.30(0.0,0.60)**
Waist circumference(cm)	**1.23(0.51,1.96)**		0.59(-0.15,1.33)	**1.09(0.37,1.81)**	**1.14(0.41,1.86)**	0.54(-0.20,1.27)
HDL-c(mmol/L)(*females*)	**-0.05(-0.08,-0.01)**	-0.03(-0.07,0.01)	-0.02(-0.06,0.02)	-0.02(-0.06,0.02)	**-0.04(-0.08,0)**	0.0(-0.03,0.03)
Triglycerides†%	**3.9(0.3,7.5)**	2.2(-1.4,5.8)	2.4(-1.4,6.2)	2.4(-1.2,6.0)	3.4(-0.2,7.0)	0.5(-3.1,4.1)
HbA$_{1c}$(%)†%	**1.2(0.4,2.0)**	**1.0(0.2,1.8)**	0.7(-0.1,1.5)	0.7(-0.1,1.5)	**1.1(0.3,1.9)**	0.4(-0.4,1.2)
**Physical abuse**						
BMI(kg/m^2)	**0.72(0.28,1.16)**		**0.71(0.27,1.14)**	**0.79(0.36,1.22)**	**0.66(0.22,1.10)**	**0.77(0.34,1.21)**
Waist circumference(cm)	**1.29(0.23,2.35)**		**1.25(0.19,2.30)**	**1.32(0.27,2.37)**	1.04(-0.02,2.10)	**1.22(0.17,2.28)**
HDL-c(mmol/L)(*females*)	**-0.06(-0.12,-0.01)**	-0.04(-0.10,0.01)	**-0.06(-0.12,-0.01)**	-0.04(-0.09,0.02)	-0.05(-0.11,0.001)	-0.01(-0.06,0.04)
HbA$_{1c}$(%)(*males*)†%	**2.5(0.7,4.3)**	**2.1(0.3, 3.9)**	**2.4(0.6,4.2)**	**1.9(0.1,3.7)**	**2.3(0.3,4.3)**	1.4(-0.4,3.2)
**Sexual abuse**						
HbA$_{1c}$(%)†%	**2.4(0.0,4.8)**	2.2(-0.2,4.6)	1.8(-0.6,4.2)	1.2(-1.2,3.6)	2.0(-0.4,4.4)	0.9(-1.5,3.3)
**Psychological abuse**						
HDL-c(mmol/L)	**-0.04(-0.07,-0.01)**	**-0.04(-0.07,-0.01)**	**-0.04(-0.07,-0.01)**	-0.02(-0.05,0.004)	-0.03(-0.06,0.001)	-0.02(-0.04,0.01)
**OR(95% CI) for elevated levels$**						
**Neglect**						
General obesity	**1.16(1.02,1.32)**		1.06(0.93,1.21)	1.13(0.99,1.29)	**1.15(1.01,1.30)**	1.05(0.92,1.20)
Central obesity	**1.15(1.02,1.30)**		1.05(0.92,1.18)	**1.13(1.00,1.27)**	**1.13(1.00,1.28)**	1.04(0.92,1.18)
**Physical abuse**						
General obesity	**1.36(1.13,1.64)**		**1.36(1.13,1.64)**	**1.38(1.14,1.66)**	**1.33(1.10,1.60)**	**1.37(1.13,1.66)**
Central obesity	**1.38(1.16,1.65)**		**1.38(1.16,1.64)**	**1.39(1.17,1.66)**	**1.34(1.12,1.60)**	**1.38(1.16,1.65)**
LDL-c	**1.25(1.00,1.56)**	1.21(0.96,1.51)	1.24(0.99,1.56)	1.16(0.93,1.46)	1.21(0.96,1.51)	1.09(0.87,1.37)
**Sexual abuse**						
LDL-c	1.41(0.89,2.23)	1.41(0.88,2.24)	1.38(0.87,2.19)	1.26(0.79,2.00)	1.34(0.84,2.13)	1.23(0.76,1.98)
**Psychological abuse**						
Triglycerides	1.21(1.02,1.44)	1.22(1.02,1.47)	1.21(1.02,1.44)	1.18(0.99,1.40)	1.15(0.96,1.37)	1.14(0.95,1.37)

NB: associations at p<0.05 (or borderline for sexual abuse) in Model 2, **Table 2** included here.
Genders combined except when gender*maltreatment interaction p≤0.05.
Model 2: model 1 factors, family history of diabetes (for HbA$_{1c}$), early-life factors (see **Table 2** footnotes)
Model 3: model 2 factors, BMI (7 and 45y).
Model 4: model 2 factors, adult social class (SEP) (own occupation at 42y (33y if missing), qualifications by 33y).

For peer review only - http://bmjopen.bmj.com/site/about/guidelines.xhtml

Model 5: model 2 factors, lifestyles at 42y (smoking, alcohol consumption, physical activity).
Model 6: model 2 factors, depressive symptoms at 42y (0-15 Malaise Inventory items).
Model 7: all variables.
†log-transformed and converted to % [NB: for HbA_{1c} and triglycerides parameters are % of units (%)]
‡all cardiometabolic markers (continuous measures) were adjusted for medication. For binary outcomes (hypertension, dyslipidemia, or T2 diabetes), those on medication were in risk groups
$details of risk groups in **Table 1**

For peer review only

For peer review only - http://bmjopen.bmj.com/site/about/guidelines.xhtml

Table 4 Associations (mean difference or OR) between number of maltreatments and cardiometabolic markers at 45y

	Number of maltreatments Per increase#
**Mean difference (95%CI)‡**	
BMI (kg/m^2)	**0.33(0.17,0.49)**
Waist circumference (cm)	
males	0.23(-0.33,0.79)
females	**1.14(0.57,1.71)**
Blood pressure	
SBP mmHg	-0.10(-0.61,0.41)
DBP mmHg	0.18(-0.17,0.53)
Blood lipids	
Total cholesterol mmol/L	0.02(-0.02,0.06)
HDL-c mmol/L	
males	-0.01(-0.03,0.01)
females	**-0.04(-0.06,-0.02)**
LDL-c	0.03(-0.01,0.07)
Triglycerides† %	**2.2(0.0,4.4)**
HbA_{1c} † %	**0.8(0.4,1.3)**
**OR (95%CI) for elevated levels$**	
General obesity	**1.13(1.05,1.21)**
Central obesity	**1.12(1.05,1.20)**
Hypertension	1.01(0.94,1.09)
HDL-c	**1.11(1.01,1.21)**
LDL-c	1.05(0.97,1.15)
Triglycerides	**1.10(1.01,1.20)**
HbA_{1c}	1.16(0.99,1.36)
Metabolic syndrome	1.10(0.96,1.26)

NB: analyses are for genders combined except where p≤0.05 for gender*maltreatment interaction where analyses are for males and females separately.

Mean difference or OR estimated from model adjusted for early life factors, including gender and factors affecting measurement (for BP: room temperature; lipids and HBA1c: examination month, time of blood collection, time since last meal, postal delay of blood sample; for females: oral contraception and HRT), plus family history of diabetes (for HbA_{1c}, diabetes) and early-life factors, including birthweight for gestational age, social class, housing tenure and crowding at 7y

†log transformed and converted to % [NB: for HbA_{1c} the parameters are % of the units (%)]

‡all cardiometabolic markers (continuous measures) were adjusted for medication. For binary outcomes (hypertension, dyslipidemia, or T2 diabetes), those on medication were in risk groups

$details of risk groups in **Table 1**

See also Table S3

For peer review only - http://bmjopen.bmj.com/site/about/guidelines.xhtml

Supplemental Table S1: Definition of child maltreatment and representative variables from the 1958 British birth cohort.

	Definition(44)	1958 British birth cohort variables*	Reference age	Age collected (Ascertainment method¥)
Neglect (prospective)‡	Failure to meet a child's basic physical, emotional, medical/dental, or education need; failure to provide adequate nutrition, hygiene, or shelter; or failure to ensure a child's safety	constructed from:<br>- child looks undernourished, scruffy or dirty<br>- hardly ever takes outings with mother<br>- hardly ever takes outings with father<br>- mother has little interest in education<br>- father has little interest in education	 7y, 11y<br><br>7y, 11y<br>7y, 11y<br>7y, 11y<br>7y, 11y	 7y, 11y(T)<br><br>7y, 11y(P)<br>7y, 11y(P)<br>7y, 11y(T)<br>7y, 11y(T)
Sexual abuse	Any completed or attempted sexual act, sexual contact, or non-contact sexual interaction with a child by a caregiver.	- I was sexually abused by a parent	0-16y	45y(S)
Physical abuse	Intentional use of physical force or implements against a child that results in, or has the potential to result in, physical injury.	- I was physically abused by a parent – punched, kicked or hit or beaten with an object, or needed medical treatment	0-16y	45y(S)
Psychological abuse	Intentional behaviour that conveys to a child that h/she is worthless, flawed, unloved, unwanted, endangered, or valued only in meeting another's needs.<br>*UK definition includes harmful (unintentional) parent-child interactions: 'the persistent emotional ill-treatment of a child such as to cause severe and persistent adverse effects on the child's emotional development'*	- I was verbally abused by a parent<br>- I suffered humiliation, ridicule, bullying or mental cruelty from a parent	0-16y	45y(S)

*for retrospective reports at 45y, information was obtained via CASI (direct computer data entry) derived from the Personality and Total Health Through Life Project (50), details of which are provided elsewhere(25). Participants were instructed: "The following are statements about your childhood. For each, please say whether the statement applies to you." Response options were: "Yes", "No", "Can't say".

¥ (S): self-report; (T): teacher-report; (P): parent-report

‡neglect (prospective) defined as ≥2 items at either 7 or 11y (note at each age, if one or two items were missing we used the sum of the remaining 4 or 3 items respectively; if >2 items were missing, we treated neglect as missing)

For peer review only

Supplemental Table S2 Associations (mean difference or OR) between childhood maltreatment and cardiometabolic biomarkers at 45y – adjusted for lifestyle factors individually and jointly

	Model 2	+adult lifestyle factors~	+smoking only	+alcohol consumption only	+physical activity only
**Mean difference (95%CI)‡**					
**Neglect**					
BMI (kg/m^2)	**0.53(0.23,0.83)**	**0.50(0.20,0.80)**	**0.60(0.31,0.90)**	**0.42(0.13,0.72)**	**0.51(0.21,0.81)**
Waist circumference (cm)	**1.23(0.51,1.96)**	**1.09(0.37,1.81)**	**1.30(0.58,2.03)**	**1.03(0.30,1.75)**	**1.18(0.46,1.90)**
HDL-c (mmol/L) (females)	**-0.05(-0.08,-0.01)**	-0.02(-0.06,0.02)	-0.03(-0.07,0.004)	-0.03(-0.07,0.004)	-0.05(-0.08,0.01)
Triglycerides† %	**3.9(0.3,7.5)**	2.4(-1.2,6.0)	2.8(-0.8,6.4)	3.5(-0.1,7.1)	**3.8(0.2,7.4)**
HbA_{1c}(%)† %	**1.2(0.4,2.0)**	0.7(-0.1,1.5)	**0.9(0.1,1.7)**	**1.0(0.2,1.8)**	**1.2(0.4,2.0)**
**Physical abuse**					
BMI (kg/m^2)	**0.72(0.28,1.16)**	**0.79(0.36,1.22)**	**0.83(0.39,1.27)**	**0.65(0.21,1.08)**	**0.73(0.30,1.17)**
Waist circumference (cm)	**1.29(0.23,2.35)**	**1.32(0.27,2.37)**	**1.38(0.32,2.44)**	**1.13(0.09,2.19)**	**1.32(0.27,2.37)**
HDL-c (mmol/L) (females)	**-0.06(-0.12,-0.01)**	-0.04(-0.09,0.02)	-0.05(-0.10,0.01)	**-0.05(-0.12,-0.01)**	**-0.06(-0.12,-0.01)**
HbA_{1c} (%) (males)† %	**2.5(0.7,4.3)**	**1.9(0.1,3.7)**	**1.9(0.1,3.7)**	**2.3(0.5,4.1)**	**2.5(0.7,4.3)**
**Sexual abuse**					
HbA_{1c}(%)† %	**2.4(0.0,4.8)**	1.2(-1.2,3.6)	1.6(-0.8,4.0)	1.9(-0.5,4.3)	2.2(-0..2,4.6)
**Psychological abuse**					
HDL-c (mmol/L)	**-0.04(-0.07,-0.01)**	-0.02(-0.05,0.004)	**-0.03(-0.06,-0.005)**	-0.03(-0.06,0.001)	**-0.04(-0.07,-0.013)**
**OR (95%CI) for elevated levels$**					
**Neglect**					
General obesity	**1.16(1.02,1.32)**	1.13(0.99,1.29)	**1.18(1.03,1.34)**	1.11(0.98,1.27)	**1.15(1.01,1.31)**
Central obesity	**1.15(1.02,1.30)**	**1.13(1.00,1.27)**	**1.16(1.03,1.31)**	**1.12(0.99,1.26)**	**1.14(1.01,1.29)**
**Physical abuse**					
General obesity	**1.36(1.13,1.64)**	**1.38(1.14,1.66)**	**1.39(1.15,1.68)**	**1.32(1.10,1.60)**	**1.37(1.14,1.65)**
Central obesity	**1.38(1.16,1.65)**	**1.39(1.17,1.66)**	**1.40(1.18,1.67)**	**1.36(1.14,1.62)**	**1.39(1.17,1.66)**
LDL-c	**1.25(1.00,1.56)**	1.16(0.93,1.46)	1.16(0.93,1.46)	1.24(0.99,1.55)	**1.25(1.00,1.57)**
**Sexual abuse**					
LDL-c	1.41(0.89,2.23)	1.26(0.79,2.00)	1.28(0.80,2.03)	1.39(0.87,2.20)	1.40(0.88,2.22)
**Psychological abuse**					
Triglycerides	**1.21(1.02,1.44)**	1.18(0.99,1.40)	1.18(0.99,1.40)	**1.20(1.01,1.43)**	**1.22(1.03,1.45)**

All cardiometabolic markers (continuous measures) were adjusted for medication. For binary outcomes, those on medication were in risk groups.
Model 2 includes all in model 1, plus family history of diabetes (for HbA_{1c}, diabetes) and early-life factors (birthweight for gestational age, social class at birth, housing tenure and crowding at 7y).
†log transformed and converted to %
‡all cardiometabolic markers (continuous measures) were adjusted for medication. For binary outcomes (dyslipidemia), those on medication were in risk groups
~shown in Table 3 as model 5: includes all in model 2, plus lifestyle factors at 42y (smoking status, alcohol consumption and physical activity.

For peer review only - http://bmjopen.bmj.com/site/about/guidelines.xhtml

Supplemental Table S3 Associations (mean difference or OR) for multiple types of maltreatment and cardiometabolic markers at 45y: unadjusted and adjusted for early-life factors

	Number of maltreatments							
	0	1		2		≥3		Per increase[#]
		Model 1	Model 2	Model 1	Model 2	Model 1	Model 2	
**Mean difference (95%CI)‡**								
BMI (kg/m^2)	-	**0.74(0.47,1.01)**	**0.47(0.19,0.74)**	**0.87(0.35,1.38)**	**0.66(0.15,1.17)**	**0.91(0.09,1.74)**	0.65(-0.18,1.47)	**0.33(0.17,0.49)**
Waist circumference (cm)								
males	-	0.74(-0.10,1.58)	0.29(-0.56,1.14)	1.13(-0.60,2.86)	0.88(-0.83,2.60)	-0.09(-3.20,3.02)	-0.46(-3.55,2.63)	0.23(-0.33,0.79)
females	-	**2.74(1.74,3.73)**	**2.19(1.17,3.21)**	**2.47(0.63,4.30)**	**2.00(0.18,3.82)**	2.32(-0.26,4.90)	1.84(-0.75,4.43)	**1.14(0.57,1.71)**
Blood pressure								
SBP mmHg	-	**1.22(0.38,2.06)**	0.70(-0.18,1.57)	-0.34(-1.96,1.28)	-0.76(-2.37,0.86)	-0.81(-3.35,1.73)	-1.53(-4.07,1.01)	-0.10(-0.61,0.41)
DBP mmHg	-	**1.01(0.43,1.59)**	**0.67(0.08,1.27)**	0.42(-0.69,1.53)	0.15(-0.96,1.25)	0.01(-1.75,1.77)	-0.45(-2.21,1.31)	0.18(-0.17,0.53)
Blood lipids								
Total cholesterol mmol/L	-	**0.06(0,0.13)**	0.05(-0.02,0.11)	0.01(-0.12,0.14)	0.01(-0.12,0.14)	0.08(-0.12,0.28)	0.07(-0.14,0.27)	0.02(-0.02,0.06)
**HDL-c mmol/L**								
**males**	-	-0.02(-0.05,0.01)	**-0.003(-0.031,0.025)**	-0.03(-0.09,0.02)	-0.02(-0.08,0.04)	-0.04(-0.15,0.07)	-0.03(-0.14,0.08)	-0.01(-0.03,0.01)
**females**	-	-0.08(-0.11,0.05)	**-0.05(-0.09,-0.02)**	-0.09(-0.15,0.02)	**-0.06(-0.13,0.0)**	-0.12(-0.21,0.03)	**-0.09(-0.18,0.0)**	**-0.04(-0.06,-0.02)**
LDL-c	-	**0.07(0.01,0.13)**	**0.06(0,0.11)**	0.04(-0.08,0.15)	0.02(-0.09,0.14)	0.09(-0.08,0.27)	0.08(-0.10,0.25)	0.03(-0.01,0.07)
Triglycerides† %	-	**6.3(2.9,9.7)**	**3.2(0,6.6)**	4.5(-1.9,10.9)	1.9(-4.5,8.3)	**10.8(0.6,21.0)**	7.4(-2.6,17.4)	**2.2(0.0,4.4)**
HbA_{1c} † %	-	**1.8(1,2.6)**	**1.4(0.6,2.2)**	**2.1(0.7,3.5)**	**1.7(0.3,3.1)**	**1.0(-1.4,3.4)**	0.5(-1.9,2.9)	**0.8(0.4,1.3)**
**OR (95%CI) for elevated levels[$]**								
General obesity	-	**1.29(1.15,1.45)**	**1.19(1.03,1.37)**	**1.39(1.11,1.73)**	**1.33(1.03,1.72)**	**1.45(1.03,2.05)**	1.37(0.93,2.00)	**1.13(1.05,1.21)**
Central obesity	-	**1.25(1.12,1.39)**	**1.16(1.04,1.29)**	**1.39(1.13,1.71)**	**1.32(1.07,1.62)**	1.26(0.91,1.74)	1.18(0.85,1.64)	**1.12(1.05,1.20)**
Hypertension	-	**1.18(1.06,1.34)**	1.11(0.98,1.26)	1.02(0.81,1.30)	0.97(0.76,1.23)	0.91(0.61,1.36)	0.84(0.56,1.25)	1.01(0.94,1.09)
HDL-c	-	**1.29(1.11,1.50)**	1.14(0.97,1.33)	**1.56(1.18,2.07)**	**1.41(1.06,1.87)**	1.17(0.74,1.85)	1.02(0.65,1.62)	**1.11(1.01,1.21)**
LDL-c	-	1.10(0.96,1.25)	1.06(0.92,1.22)	1.17(0.89,1.54)	1.14(0.86,1.50)	1.16(0.76,1.78)	1.12(0.73,1.72)	1.05(0.97,1.15)
Triglycerides	-	**1.24(1.09,1.40)**	**1.14(1.00,1.30)**	1.24(0.96,1.59)	1.16(0.90,1.50)	1.41(0.94,2.10)	1.30(0.87,1.94)	**1.10(1.01,1.20)**
HbA_{1c}	-	**1.47(1.15,1.87)**	**1.31(1.01,1.69)**	1.47(0.89,2.43)	1.33(0.80,2.21)	1.30(0.54,3.18)	1.12(0.46,2.74)	1.16(0.99,1.36)
Metabolic syndrome	-	**1.26(1.03,1.54)**	1.15(0.95,1.39)	1.37(0.94,1.99)	1.27(0.87,1.83)	1.15(0.61,2.16)	1.06(0.57,1.96)	1.10(0.96,1.26)

NB: analyses are for genders combined except where p≤0.05 for gender*maltreatment interaction where analyses are for males and females separately.

[#] per increase based on Model 2

Model 1 includes gender and factors affecting measurement (room temperature for BP; month of examination, time of day of blood collection, time since last meal, postal delay of blood sample for lipids and HbA_{1c}; for females oral contraception and HRT for all outcomes). All factors were included for metabolic syndrome

For peer review only

For peer review only - http://bmjopen.bmj.com/site/about/guidelines.xhtml

Model 2 includes all in model 1, plus family history of diabetes (for HbA_{1c}, diabetes) and early-life factors, including birthweight for gestational age, social class, housing tenure and crowding at 7y
†log transformed and converted to % [NB: for HbA_{1c} the parameters are % of the units (%)]
‡all cardiometabolic markers (continuous measures) were adjusted for medication. For binary outcomes (hypertension, dyslipidemia, or T2 diabetes), those on medication were in risk groups
$details of risk groups in **Table 1**

For peer review only

BMJ Open

Childhood maltreatment and biomarkers for cardiometabolic disease in mid-adulthood in a prospective British birth cohort: associations and potential explanations

Journal:	*BMJ Open*
Manuscript ID	bmjopen-2018-024079.R1
Article Type:	Research
Date Submitted by the Author:	31-Oct-2018
Complete List of Authors:	Li, Leah; UCL Institute of Child Health, Population Policy and Practice Programme<br>Pinto Pereira, Snehal; University College London Institute of Child Health, Population, Policy and Practice<br>Power, Christine
**Primary Subject Heading**:	Cardiovascular medicine
Secondary Subject Heading:	Epidemiology, Paediatrics, Public health
Keywords:	child maltreatment, child abuse, child neglect, cardiometabolic biomarkers, cohort study

SCHOLARONE™ Manuscripts

TITLE PAGE

Childhood maltreatment and biomarkers for cardiometabolic disease in mid-adulthood in a prospective British birth cohort: associations and potential explanations

Leah Li (leah.li@ucl.ac.uk)*, Snehal M Pinto Pereira (snehal.pereira@ucl.ac.uk), Chris Power (christine.power@ucl.ac.uk)

Population, Policy, and Practice Programme, University College London Great Ormond Street Institute of Child Health, 30 Guilford Street, London WC1N 1EH, UK.

*** Corresponding author**

For peer review only

Abstract

Objectives: Research on associations between childhood maltreatment and adult cardiometabolic disease risk is sparse. We aimed to investigate associations between different forms of child maltreatment and mid-adult cardiometabolic markers and whether potential intermediaries could account for the associations observed.

Setting: 1958 British birth cohort.

Participants: Approximately 9000 cohort members with data on cardiometabolic markers.

Outcomes: Adult (45y) cardiometabolic markers (blood pressure, lipids, glycated haemoglobin (HbA_{1c})).

Results: Seventeen percent of participants were identified as neglected, 6.1%, 1.6%, and 10.0% as experiencing physical, sexual and psychological abuse respectively. Childhood neglect and physical abuse were associated with high BMI and waist circumference when adjusting for early-life covariates. For neglect, the adjusted odds ratio (AOR) was (1.16(95% CI:1.02,1.32)) for general obesity and 1.15(1.02,1.30) for central obesity, and for physical abuse the respective AOR was 1.36(1.13,1.64) and 1.38(1.16,1.65). Neglect was also associated with raised triglycerides by 3.9(0.3,7.5)% and HbA_{1c} by 1.2(0.4,2.0)%, and, among females, lower HDL-c by 0.05(0.01,0.08)mmol/L after adjustment. For physical abuse, the AOR was 1.25(1.00,1.56) for high LDL-c, HbA_{1c} was raised by 2.5(0.7,4.3)% (in males) and HDL-c was lower by 0.06(0.01,0.12)mmol/L (in females)). Associations for sexual abuse were similar to those for physical abuse but 95% CIs were wide. For psychological abuse the AOR for elevated triglycerides was 1.21(1.02,1.44) and HDL-c was lower by 0.04(0.01,0.07)mmol/L. Maltreatments were not associated with raised blood pressure. In analyses of potential intermediary factors, several associations attenuated after adjustment for adult lifestyles (mainly smoking and alcohol consumption rather than physical activity) and child-to-adult BMI.

Conclusions: Childhood maltreatments, particularly neglect and physical abuse, were associated with greater adiposity and poorer lipid and HbA_{1c} profiles decades later in adulthood. Associations were modest, but independent of early-life factors linked to these outcomes. Findings implicate adult lifestyles as an important intermediary between child maltreatment and outcomes.

Key words: child maltreatment, child abuse, child neglect, cardiometabolic biomarkers, cohort study

For peer review only - http://bmjopen.bmj.com/site/about/guidelines.xhtml

Strengths and limitations of this study

- Data were from a population-based cohort followed-up over five decades and included information on a range of adult cardiometabolic measures, childhood maltreatment (neglect and abuse), childhood covariates, and potential adult intermediary factors.
- Sample reductions due to missing information were addressed using multiple imputation.
- Study power for sexual abuse may be inadequate for detecting associations due to low prevalence.

For peer review only

For peer review only - http://bmjopen.bmj.com/site/about/guidelines.xhtml

Background

There is now evidence showing links between early-life adversities and later cardiovascular disease (CVD) incidence and mortality(1-3), including some with measures of child maltreatment (neglect and abuse)(4-8). In the US adverse childhood experiences (ACE) study, reported child neglect or abuse was found to be associated with increased risk of self-reported ischemic heart disease (odds ratio (OR)=1.3 to 1.7 for specific forms of maltreatment); the highest risk (OR=3.6 (95% CI: 2.4,5.3) was found for multiple ACEs (≥7 vs 0) (5) as seen also in a more recent US study (6). Among 33-45 year-olds in the Coronary Artery Risk Development in Young Adults Study, a 1-unit increase in risky family score (range 0-21 from self-report items) was associated with 1.0% higher 10-year CHD risk (using the Framingham algorithm)(7). Mortality follow-up studies have shown self-reported child abuse to predict mortality from all-causes in the US (9), whilst those with severe (vs. no) physical abuse had increased risk for CVD events (hazard ratio (HR)= 1.46 (1.11,1.92)) in the Nurses Health Study (8). Accordingly, a scientific statement from the American Heart Association identified severe childhood adversities such as physical and sexual abuse as emerging independent risk factors for incident ischemic heart disease among women(10).

However, much remains unknown about child maltreatment and CVD risk associations, including whether these are specific to different forms of maltreatment, whether associations are independent of other early-life factors and what the likely explanations or pathways are. Relevant to the latter is the growing evidence on child maltreatment and CVD risk factors, which provides important clues on possible intermediary pathways to CVD events. Investigation of a range of cardiometabolic biomarkers would be informative, but studies to date typically focus on blood pressure (BP)(11-14) or obesity(15), and rarely include blood lipids(7;16;17) or glucose metabolism(18-20). Study findings are not always consistent: e.g., some(11) but not all(12) show associations of physical or sexual abuse with raised BP. Differences in study design may account for such discrepancies. In particular, it is important to account for other early-life factors, such as low birthweight(1) and socio-economic position (SEP)(2) linked to these outcomes. Moreover, the literature mostly focuses on child abuse(11;12;19;21) whist neglect is often only included as a component of a combined measure (5;18;22) despite its association with increased adult body mass index (BMI)(23), a risk factor for cardiometabolic disease. Combined maltreatment measures may be useful to demonstrate cumulative effects

For peer review only - http://bmjopen.bmj.com/site/about/guidelines.xhtml

but potentially obscure important differences that provide insights into mechanisms. Thus, two recent meta-analyses of long-term health consequences of non-sexual maltreatment(3) or cumulative childhood adversity(4), concluded that research is required to confirm relationships between all forms of child maltreatment, including neglect, and later chronic disease.

With respect to explanations or pathways, notwithstanding a few exceptions (21), research is scarce on factors underlying the associations between forms of child maltreatment and biomarkers for adult cardiometabolic disease. Yet potential mechanisms can be identified, such as allostatic load, whereby physiological wear-and-tear over the life course (24) may occur in response to child maltreatment and thence influence adult health. Several intermediary factors may be postulated. First, associations may be due to greater BMI gains (21;25) or obesity (11;26) among maltreated groups. Second, child maltreatment may influence adult SEP (27), e.g. via poorer education level (28), which in turn affects adult health. Third, maltreatment may lead to risky behaviours (e.g. smoking (29) or alcohol use (30)) which impact on disease risk. Fourth, the influence of maltreatment on mental health problems including depression (28), could link to cardiometabolic biomarkers via increased physiologic response to stressors (31) or via unhealthy behaviours (3). We examined these potential intermediaries in a general population birth cohort. Our study aims were to investigate (i) whether different forms of childhood maltreatment (neglect and abuse) were associated with adult cardiometabolic measures (obesity, BP, blood lipids and glycated haemaglobin) independent of other early-life factors such as birthweight and SEP; and (ii) whether intermediary factors (life-course BMI gains, adult SEP, unhealthy behaviours, poor mental health) could account for associations.

Methods

The 1958 British birth cohort includes all births during one week March 1958 (N=17,638) and 920 immigrants with the same birth week recruited to 16y. Information was collected throughout childhood(7, 11, 16y) and adulthood(23, 33, 42, 45, 50, 55y). At 45y, 11,971 individuals in contact with the study were invited to a home-based clinical assessment; 9,377 (78%) participants who provided information were broadly representative of the surviving cohort (32). The 45y survey included a childhood maltreatment

For peer review only - http://bmjopen.bmj.com/site/about/guidelines.xhtml

questionnaire, to which participants gave written informed consent. Ethical approval was given by the South-East Multi-Centre Research Ethics Committee (ref:01/1/44).

Childhood maltreatment: Information was ascertained during childhood and at 45y (**Table S1**). Neglect was prospectively assessed (at ages 7 and 11y) using five questions to parents on their involvement with the child (infrequent outings and little interest in education) and to teachers (child's physical appearance undernourished, scruffy or dirty) that correspond to conventional definitions (details in **Table S1**). A scale (range 0-5) was derived by summing the five indicators separately for each age and a binary measure derived as ≥2 items (7y and/or 11y). Child abuse (physical, sexual or psychological) by a parent up to 16y (yes/no) was reported in adulthood (45y) in a confidential self-complete questionnaire (27) (details in **Table S1**).

*Outcomes***:** cardiometabolic measurements were obtained at 45y by nurses using standardized protocols. Height, weight and waist circumference were measured; BMI was derived as kg/m^2. BP was measured three times while seated after five mins rest using an Omron 705CP automated digital oscillometric sphygmomanometer; means of three systolic BP (SBP) and diastolic BP (DBP) measurements were calculated. Non-fasting venous blood samples were collected and analysed for total and high density lipoprotein cholesterol (HDL-c) and triglyceride concentrations using an autoanalyzer (Olympus AU640; Tokyo) with enzymatic methods. Low density lipoprotein cholesterol (LDL-c) was calculated using Friedewald's formula (33) when triglyceride concentration<4.5mmol/L. Glycated hemoglobin (HbA_{1c}) concentrations were measured in whole citrated blood using ion-exchange HPLC. Nurses recorded information on prescribed medications: anti-hypertensive (N=429), lipid-regulating (N=166), oral glucose-lowering/antidiabetic (type 2 (T2) diabetes) (N=111); T2 diabetes was also identified from self-reported doctor diagnosis at 42y (N=134) excluding type 1 diabetes (N=57).

Cardiometabolic biomarkers (binary variables) were derived using established cut-offs (**Table 1**) from the literature(34;35) and our previous studies (36). These include general obesity (BMI≥30kg/m²) and central obesity (waist circumference ≥102cm (males), ≥88cm (females)(34)), hypertension (SBP/DBP≥ 140/90mmHg or anti-hypertensive medication(37)), high triglycerides (>2.3mmol/L(38)), low HDL-c (≤1.0mmol/L (males), ≤1.3mmol/L (females)), and high LDL-c (>4.13mmol/L(35)). High triglycerides, low

For peer review only - http://bmjopen.bmj.com/site/about/guidelines.xhtml

HDL-c, and high LDL-c groups also include individuals on lipid-regulating medications. Elevated (≥6%) HbA_{1c} (39), and T2 diabetes were grouped together, excluding T1 diabetes. Metabolic syndrome was defined using modified criteria of the Third Report of the National Cholesterol Education Program's Adult Treatment Panel (35) as ≥3 of: central obesity, high BP, low HDL-c, high triglycerides, and elevated HbA_{1c} (or T2 diabetes) as defined above.

Covariates: were selected a-priori from the literature based on relationships with child maltreatment and adult cardiometabolic biomarkers, including factors affecting (measurement of) outcomes, early-life factors, and potential intermediary factors. Early-life factors were all recorded prospectively at birth (birthweight for gestational age, childhood social class) or at age 7y (household crowding and housing tenure). Birthweight for gestational age was calculated as birthweight (measured) standardized within each gestational week. Social class was based on father's occupation at birth (or at 7y if missing), classified as I/II (professional/managerial); IIINM (skilled non-manual); IIIM (skilled manual); IV/V (semi-unskilled manual, including no male head). Overcrowding (≥1.5 persons per room) and social housing (rented from the council or housing association, versus owner-occupied or privately rented) were used as measures of material (dis)advantage.

Factors affecting (measurement of) outcomes at 45y included: measured room temperature (for BP), month of examination, time of day of blood collection, postal delay of blood sample, self-reported time since last meal (for lipids and HbA_{1c}), and oral contraception (10.8%) and HRT use (7.4%) for females (for all outcomes), self-reported family history of T2 diabetes (2.4%) (for HbA_{1c}). All factors affecting measurement of the outcome were included in the analysis of metabolic syndrome.

Intermediary factors for associations between maltreatment and cardiometabolic markers included childhood (7y) and adult (45y) BMI, adult SEP (social class, educational qualifications), adult lifestyles (smoking, alcohol consumption, physical activity) and mental health as indexed by depressive symptoms. BMI was derived from measured heights and weights at ages 7y and 45y. Adult social class was based on the participant's occupation at 42y (or at 33y if missing). Educational qualifications attained by 33y, was categorised into: degree/higher; A-level; O-level; <O-level; no qualifications. Smoking status was reported at

For peer review only - http://bmjopen.bmj.com/site/about/guidelines.xhtml

42y (or at 33y if missing). Alcohol consumption frequency was reported at 42y, classified as: non-drinker; ≤once/month; ≤3 time/month; once/week; 2-3 days/week; most days. Leisure-time physical activity, ascertained at 42y from questions on how often cohort members participated in a range of activities, was categorised as: active≤3 times/month; 1 day/week; 2–3 days/week; 4–7 days/week(40). For depressive symptoms at 42y a 0-15 scale was derived from 15 psychological items included in the Malaise Inventory(41).

Analysis

Associations of each child maltreatment with continuous outcomes (measured by differences in mean levels) and binary outcomes (measured by odds ratios (ORs)) were estimated using linear and logistic regressions respectively, for genders combined, except where associations differed ($P \leq 0.05$ for gender*maltreatment interaction) which were analysed separately. Continuous triglycerides and HbA_{1c} measures were log-transformed to correct skewness of distributions; % change in mean level approximates 100*(regression coefficient)(42). Models included corrections for effects of medication to minimise potential bias in estimates: (i) for antihypertensive medication (N=429) we added a commonly used constant of 10mmHg (which has been suggested on the basis of evidence from clinical trials) to observed SBP and DBP (43); (ii) lipid levels (N=166) were corrected assuming that lipid-lowering drugs reduce total cholesterol by 20%, LDL-c by 35%, triglycerides by 15% and increase HDL-c by 5%, based on average efficacy of statins, the most frequently prescribed lipid-lowering drug in this study (44); (iii) for antidiabetic medication (N=111) HbA_{1c} levels were corrected assuming that medication reduced HbA_{1c} levels by 1% in absolute terms (45).

Baseline models included gender and factors affecting measurements (model 1). Models were then adjusted for early-life factors (and, for HbA_{1c}, family history of T2 diabetes) (model 2) and adjusted differences in mean levels and adjusted OR (AOR) were estimated. For associations that remained in model 2 ($P<0.05$, or borderline for sexual abuse due to low prevalence), we further adjusted for potential intermediary factors. To test the separate and combined intermediary role of these factors, we examined models that further adjusted for: (1) BMI at 7 and 45y, (2) SEP (social class, educational qualifications), (3) lifestyle factors (smoking, alcohol consumption, physical activity), (4) depressive symptoms, and (5) all factors simultaneously (models

For peer review only - http://bmjopen.bmj.com/site/about/guidelines.xhtml

3-7). However, conventional regression models with adjustment for potential mediators could possibly induce bias e.g., via exposure-mediator interactions(46). Hence, we conducted selected checks to ensure that associations were unaffected by such biases: (i) tests of maltreatment-intermediary factor interaction(s) which were mostly non-significant; (ii) adjustments to test mediator-outcome confounding: we examined effects on parameter estimates of additional adjustment for adult SEP which is associated with both intermediary factors (e.g. lifestyles) and cardiometabolic outcomes(44); effects of such adjustments were negligible. As an additional check we conducted mediation analysis using Inverse Odds Ratio Weighting method(47). General patterns of mediation effects were consistent with those obtained from regression models 3-7 and conclusions remained unchanged. Hence we present results using conventional regression with adjustment for mediators for simplicity of interpretation.

In addition to analyses of specific types, we examined cumulative burden of maltreatment, as in previous research(5); we summed the number of types of maltreatment and, using linear regression, examined associations with cardiometabolic measures, with and without adjustment for early-life factors.

As sensitivity analyses, we examined effects of treatments (i) with adjustment for medications rather than using correction and (ii) excluding those who were on treatment. Results were similar to those presented using correction for treatment (data not shown).

Among participants with cardiometabolic markers (N=7391-9297 depending on outcome), 93% had information on neglect and nearly all on abuse. To maximise available information, we applied multiple imputation to missing data on covariates and neglect to the sample alive to 45y (N=17313). Imputation models included analysis variables (maltreatment, cardiometabolic markers, covariates) and predictors of non-response(32) (gender, ethnicity, class at birth, 7y reading ability). We created 25 imputed datasets assuming missing at random given other variables in the imputation models. Analysis samples were restricted to individuals with observed cardiometabolic markers and abuse measures (analysis sample N=7391-9297). Parameters from imputed datasets were combined to obtain overall estimates using Rubin's rules(48). Regression models and multiple imputation by chained equations were conducted using SPSS (Version 24.0). Additional mediation analysis was conducted using STATA (version 15.0).

For peer review only - http://bmjopen.bmj.com/site/about/guidelines.xhtml

Patient and public involvement

Patients and public were not involved in the design of the study.

Results

Approximately 12.0% of participants reported any childhood abuse: 6.1% (physical), 1.6% (sexual), and 10.0% (psychological) abuse, 17.4% were classified as neglected; most were not maltreated, 20.4% had one and 6.1% had ≥2 types (**Table 1**).

Childhood neglect was associated with several cardiometabolic biomarkers after adjusting for factors affecting measurement and early-life covariates: BMI and waist circumference were higher by 0.53(95% CI:0.23,0.83)kg/m^2 and 1.23(0.51,1.96)cm respectively, AORs were higher for both general (1.16(1.02,1.32)) and central 1.15(1.02,1.30) obesity; triglycerides were raised by 3.9(0.3,7.5)% and HbA_{1c} by 1.2(0.4,2.0)%, and, among females, HDL-c was lower by 0.05(0.01,0.08)mmol/L (**Table 2**). When considering potential intermediary factors, associations reduced (mostly disappeared) after further adjusting for child-to-adult BMI, adult SEP and lifestyles (mainly smoking and alcohol consumption rather than physical activity) (**Tables 3** and **S2**).

Physical abuse was associated with higher BMI (by 0.72(0.28,1.16) kg/m^2), waist circumference (by 1.29(0.23,2.35)cm) and obesity (AOR=1.36(1.13,1.64) for general obesity; 1.38(1.16,1.65) for central obesity); increased risk of high LDL-c (AOR=1.25(1.00,1.56)), raised HbA_{1c} in males (by 2.5(0.7,4.3)%), and lower HDL-c in females (by 0.06(0.01,0.12)mmol/L) (**Table 2**). In relation to potential intermediary factors, associations largely attenuated when adjusting for adult lifestyles (notably smoking), and child-to-adult BMI (for HDL-c in females) except for BMI, waist circumference and obesity which were unaffected (**Tables 3** and **S2**). *Sexual abuse* associations with biomarkers were similar or greater in magnitude than other maltreatments, e.g. physical abuse, but CIs were wide due to few cases (**Table 2**): e.g., AOR of high LDL-c was 1.41(0.89,2.23) after adjustment for early-life factors, attenuating to 1.26(0.79,2.00) when

For peer review only - http://bmjopen.bmj.com/site/about/guidelines.xhtml

adjusting for lifestyles, predominantly smoking (**Tables 3** and **S2**). *Psychological abuse* was associated with elevated risk of high triglyceride levels (AOR=1.21(1.02,1.44)) and low HDL-c (by 0.04(0.01,0.07)mmol/L) (**Table 2**). When considering potential intermediary factors, associations disappeared when adjusting for lifestyles (mostly smoking) and mental health (**Tables 3** and **S2**).

Childhood maltreatments were not associated with raised BP or risk of metabolic syndrome, but since prevalence is low for sexual abuse we cannot rule out elevated risks for either metabolic syndrome or T2 diabetes (**Table 2**). Intriguingly, sexual abuse was associated with a reduced risk for hypertension (AOR=0.43(0.24,0.74)); additional analysis for pulse pressure showed similar findings (results not presented). Whereas for other maltreatments, the null findings for BP, hypertension, metabolic syndrome or T2 diabetes are less likely to be due to lack of statistical power. Finally, few associations were found between number of types of maltreatment and cardiometabolic outcomes after adjusting for early-life factors; exceptions included BMI and elevated triglyceride levels, the latter reflecting weak and mostly non-significant associations across all maltreatment types (**Table 4** and **S3**).

Discussion

Main findings include, first, all forms of child maltreatment were associated with at least one adverse cardiometabolic outcome (adiposity, blood lipids and/or HbA_{1c}) in mid-adulthood, although there was no evidence of adverse effects on BP. Importantly, associations were independent of birthweight and early-life SEP shown elsewhere to be associated with biomarkers for cardiometabolic disease(1;2). Associations were consistent for neglect and physical abuse in relation to adiposity, whilst for other maltreatments and outcomes associations were few, effects were modest, and evidence was weak for accumulative associations for number of types of maltreatment. Second, in relation to potential intermediary factors for the child maltreatment-cardiometabolic associations observed, these mostly disappeared after adjusting for adult lifestyles, suggesting that lifestyles may play a key mediating role, whilst adult SEP was important for the association of neglect but not physical abuse with adiposity.

Methodological considerations

For peer review only - http://bmjopen.bmj.com/site/about/guidelines.xhtml

Study strengths include a population-based cohort followed-up over five decades, with a range of adult cardiometabolic measures, prospectively ascertained childhood covariates including multiple indicators of family SEP, and potential intermediary factors. Measurement of childhood neglect was recorded prospectively from multiple sources; items relate to some dimensions (failure to meet a child's basic physical, emotional and education needs) but not all aspects (e.g. inadequate nutrition or shelter) of neglect(49). As individual items may not imply neglectful behaviour, we used a score of ≥two items. Such measures are associated with delayed childhood height growth(50), cognitive ability and poorer qualifications(28) in this population, as expected from the wider literature using differing study designs and thereby supporting construct validity for our neglect measures. Childhood abuse was self-reported at 45y. All ascertainment methods for child maltreatment have limitations(49). Parental report may be influenced by socially desirable responding and miss cases due to under-reporting, likewise only a small proportion of cases are identified by agencies. Whilst adult report of abuse may be affected by recall bias or current emotional state(51), retrospective report provides an accepted method in population studies(49); in the 1958 cohort it was blind to knowledge of issues to be investigated and data show expected associations with prospectively measured family dysfunction(52) and mental health(28), suggesting good construct validity. Moreover, it has been suggested that retrospective reports show less biased associations with objective adult outcomes (such as the risk factors for cardiometabolic disease examined here) than self-reported outcomes(53). Some cardiometabolic measures were collected from non-fasted blood. Such measures may be inappropriate for clinical purposes (triglycerides levels tend to be lower in non-fasted samples(54) and vary by fasting duration and time of day(55)) but are often adequate for population studies given that fasting and non-fasting levels (e.g. of triglycerides) are positively correlated(56) and because non-fasting levels have been found to be a significant risk factor for cardiovascular disease(57;58). Moreover, misclassification of lipid or triglyceride levels by our exposures is unlikely, and we adjusted for time of last meal and blood collection in analyses for lipids and HbA_{1c}. As in other longitudinal studies, sample attrition had occurred over time. Although respondents in mid-adulthood were generally representative of the original cohort (32), our previous study showed that individuals with childhood adversities (e.g. neglect) were more likely than others to be lost to follow-up at 45y and thus are under-represented in the present study(52). Although the possibility of attrition bias cannot be ruled out, our previous work on child neglect associations with other adult outcomes suggests that its effect is likely to be negligible(28), as seen also in further investigation of

For peer review only - http://bmjopen.bmj.com/site/about/guidelines.xhtml

attrition bias in relation to glucose measures at 45y(59). Sample reductions due to missing covariates and neglect measures were addressed using multiple imputation. Associations between types of maltreatment and outcomes estimated from imputed data (i.e. amongst the sample with data on outcomes at 45y) were broadly similar to those obtained from samples with observed data (data not shown). Finally, study power for sexual abuse may be inadequate for detecting associations due to low prevalence. Regarding methodological approach to assess potential intermediary factors for maltreatment-cardiometabolic associations, we used conventional regression and confirmed the robustness of our conclusions using additional checks (including Inverse Odds Ratio Weighting method(47)).

Interpretation and comparison with other studies

Our main finding of few modest associations between types of child maltreatment and biomarkers for cardiometabolic disease adds to the literature in several important respects. First, the adverse associations found for adult adiposity, blood lipids and HbA_{1c} but not BP, suggests the primary biological pathways through which some maltreatments could have deleterious effects on later cardiometabolic morbidity and mortality. These findings agree with others in their emphasis on adiposity(15) and lipids/glucose(18-20), and whilst several studies report associations with BP(11;13;17), not all do(12;16). One possibility is that some BP findings reported to date are conflated by lack of control for other early-life factors, a short-coming that we have been able to address. Moreover, others suggest, as we do, that associations are small to modest(7). Similar to our findings, the CARDIA study reported that ‘risky’ family environment (cold/unaffectionate family interactions, conflict, aggression, neglect, low nurturance) was associated with HDL-c level in women, but not with BP(7). Specific types of maltreatment in our study, namely neglect and physical abuse, appeared to be particularly relevant to cardiometabolic outcomes, although as mentioned above associations for sexual abuse were comparable in magnitude. Comparison with existing research is hindered by the dearth of studies that distinguish neglect from other maltreatments, but our associations of neglect and physical abuse with increased HbA_{1c} (potentially T2 diabetes) agree with evidence to date(18-20) and results for physical/sexual abuse are consistent with an increased CVD risk for women(8). Unlike others(5), we found little evidence of associations for number of types of maltreatment, a null finding that supports the suggestion of there being a few specific associations rather than cumulation across several types of

For peer review only - http://bmjopen.bmj.com/site/about/guidelines.xhtml

maltreatment. Our findings contrast with previous research in other respects, e.g., the lack of association for non-sexual abuse and metabolic syndrome (despite associations for key lipid and glucose components) contrasts with reported increased risk for physical abuse(16;21). Such discrepancies may arise from our null findings for BP and other considerations such as differences in design (e.g. self-reported hypertension(11;13)) or in age(13;17). Associations for maltreatment may vary by age, emerging later in life, as suggested by studies showing rapid gains in BP(14) and in BMI from early adulthood(25).

For our second main finding, results suggest that adult lifestyle was a key proximal intermediary factor for several maltreatment–biomarker associations, which concurs with some(5;7;8;60), though not all(11;19) previous studies. Our interpretation of this finding is that it suggests child neglect and abuse are associated with higher rates of smoking and obesity(11;25;29) that, in turn, affect cardiometabolic markers such as lipids and HbA_{1c}. Concordant with our results, the US Nurses Health Study found that adult lifestyle and medical risk factors accounted for much of the association between child abuse and adult CVD(8). In relation to other potential explanations, we would expect adult SEP to be an important factor, as reported elsewhere(61), especially for neglect rather than non-sexual abuse given its association with lower adult SEP in this cohort(27). Our results confirm this expectation, as several associations for neglect (less so for abuse) attenuated after allowing for adult SEP. Of the other hypothesised intermediary factors examined, child-to-adult BMI trajectory appeared to contribute to associations for neglect and physical abuse with adult lipid and HbA_{1c} levels. This agrees with the US Nurses Health Study in which adult BMI was found to account for most of the associations between physical/sexual abuse and adult T2 diabetes(19). Lastly for mental health, the suggestion in our study that this was an important pathway for psychological abuse is unsurprising. We might have expected it to impact other maltreatment- biomarker associations but found only negligible effects, although psychological processes other than depressive symptomatology could contribute.

Conclusions and implications

Childhood maltreatments, particularly neglect and physical abuse, were associated with greater adiposity and poorer lipid and HbA_{1c} profiles in mid-adulthood, suggesting further long-term health consequences for these groups. Although associations were only modest, importantly, they were independent of main early-life

For peer review only - http://bmjopen.bmj.com/site/about/guidelines.xhtml

factors (birthweight, SEP) linked previously to these outcomes. Study findings suggest that adult lifestyles and child-to-adult BMI may be key intermediaries for many associations, with some evidence for additional factors, such as adult SEP. The role of child maltreatment for smoking and adiposity as intermediaries linked to later disease is relevant to clinicians and policy-makers aiming to reduce later consequences associated with child maltreatment. However, whether these intermediary risk factors among child maltreatment groups would benefit from standard interventions (e.g. for smoking cessation) in adulthood remains to be answered. Such interventions may be ineffective, for example, the propensity to smoke or to gain BMI is biologically embedded from an early age and is difficult to alter thereafter. This would strengthen further the need to develop strategies for preventing childhood maltreatment (e.g. early home visiting or parenting programmes targeting those at risk for child abuse and neglect). Further work is needed on the role of life-course intermediary factors to better understand the pathways and mechanisms underlying maltreatments-cardiometabolic disease associations, including consideration of differing as well as common pathways for specific maltreatments. These efforts are essential to effective strategies to reduce or prevent adult health consequences of child maltreatment.

Conflict of interest: None to declare.

Abbreviations:

Cardiovascular disease (CVD)

Adverse childhood experiences (ACE)

Odds ratio (OR)

Blood pressure (BP)

Socio-economic position (SEP)

Body mass index (BMI)

High density lipoprotein cholesterol (HDL-c)

Low density lipoprotein cholesterol (LDL-c)

Glycated haemoglobin (HbA_{1c})

For peer review only - http://bmjopen.bmj.com/site/about/guidelines.xhtml

Ethics approval and consent to participate: The 45y survey included a childhood maltreatment questionnaire, to which participants gave written informed consent. Ethical approval was given by the South-East Multi-Centre Research Ethics Committee (ref:01/1/44).

Consent for publication: Not applicable.

Availability of data and material

NCDS data used in this research are available from the UK Data Archive (http://www.data-archive.ac.uk); applications for access to any data that forms part of the NCDS Biomedical Resource will require special license and should be submitted to clsfeedback@ioe.ac.uk.

Funding sources

This work was funded by the Department of Health Policy Research Programme through the Public Health Research Consortium (PHRC) and supported by the National Institute for Health Research Biomedical Research Centre at Great Ormond Street Hospital for Children NHS Foundation Trust and University College London. The views expressed in the publication are those of the authors and not necessarily those of the Department of Health. Information about the wider programme of the PHRC is available from http://phrc.lshtm.ac.uk. Data collection for participants at age 45 was funded by the Medical Research Council, grant G0000934. The authors are grateful to the Centre for Longitudinal Studies (CLS), University College London Institute of Education for the use of these data and to the UK Data Service for making them available. However, neither CLS nor the UK Data Service bears any responsibility for the analysis or interpretation of these data.

Author contributions

LL conceptualised the study, carried out the analyses, drafted the manuscript, revised it critically for important intellectual content and approved the final version to be published.

CP conceptualised the study, drafted the manuscript, revised it critically for important intellectual content and approved the final version to be published.

For peer review only - http://bmjopen.bmj.com/site/about/guidelines.xhtml

SMPP revised the paper critically for important intellectual content and approved the final version to be published.

For peer review only

For peer review only - http://bmjopen.bmj.com/site/about/guidelines.xhtml

Reference List

(1) Huxley R, Owen CG, Whincup PH, Cook DG, Colman S, Collins R. Birth weight and subsequent cholesterol levels: exploration of the "fetal origins" hypothesis. JAMA 2004;292(22):2755-64.

(2) Power C, Kuh D, Morton S. From developmental origins of adult disease to life course research on adult disease and aging: insights from birth cohort studies. Annu Rev Public Health 2013;34:7-28.

(3) Norman RE, Byambaa M, De R, Butchart A, Scott J, Vos T. The long-term health consequences of child physical abuse, emotional abuse, and neglect: a systematic review and meta-analysis. PLoS Med 2012;9(11):e1001349.

(4) Jakubowski KP, Cundiff JM, Matthews KA. Cumulative childhood adversity and adult cardiometabolic disease: A meta-analysis. Health Psychol 2018;37(8):701-15.

(5) Dong M, Giles WH, Felitti VJ, Dube SR, Williams JE, Chapman DP, et al. Insights into causal pathways for ischemic heart disease: adverse childhood experiences study. Circulation 2004;110(13):1761-6.

(6) Gilbert LK, Breiding MJ, Merrick MT, Thompson WW, Ford DC, Dhingra SS, et al. Childhood adversity and adult chronic disease: an update from ten states and the District of Columbia, 2010. Am J Prev Med 2015;48(3):345-9.

(7) Loucks EB, Almeida ND, Taylor SE, Matthews KA. Childhood family psychosocial environment and coronary heart disease risk. Psychosom Med 2011;73(7):563-71.

(8) Rich-Edwards JW, Mason S, Rexrode K, Spiegelman D, Hibert E, Kawachi I, et al. Physical and sexual abuse in childhood as predictors of early-onset cardiovascular events in women. Circulation 2012;126(8):920-7.

(9) Chen E, Turiano NA, Mroczek DK, Miller GE. Association of Reports of Childhood Abuse and All-Cause Mortality Rates in Women. JAMA Psychiatry 2016;73(9):920-7.

(10) Mehta LS, Beckie TM, DeVon HA, Grines CL, Krumholz HM, Johnson MN, et al. Acute Myocardial Infarction in Women: A Scientific Statement From the American Heart Association. Circulation 2016;133(9):916-47.

(11) Riley EH, Wright RJ, Jun HJ, Hibert EN, Rich-Edwards JW. Hypertension in adult survivors of child abuse: observations from the Nurses' Health Study II. J Epidemiol Community Health 2010;64(5):413-8.

(12) Gooding HC, Milliren C, McLaughlin KA, Richmond TK, Katz-Wise SL, Rich-Edwards J, et al. Child maltreatment and blood pressure in young adulthood. Child Abuse Negl 2014;38(11):1747-54.

(13) Stein DJ, Scott K, Haro Abad JM, Aguilar-Gaxiola S, Alonso J, Angermeyer M, et al. Early childhood adversity and later hypertension: data from the World Mental Health Survey. Ann Clin Psychiatry 2010;22(1):19-28.

(14) Su S, Wang X, Pollock JS, Treiber FA, Xu X, Snieder H, et al. Adverse childhood experiences and blood pressure trajectories from childhood to young adulthood: the Georgia stress and Heart study. Circulation 2015;131(19):1674-81.

(15) Noll JG, Zeller MH, Trickett PK, Putnam FW. Obesity risk for female victims of childhood sexual abuse: a prospective study. Pediatrics 2007;120(1):e61-e67.

For peer review only

(16) Midei AJ, Matthews KA, Chang YF, Bromberger JT. Childhood physical abuse is associated with incident metabolic syndrome in mid-life women. Health Psychol 2013;32(2):121-7.

(17) McIntyre RS, Soczynska JK, Liauw SS, Woldeyohannes HO, Brietzke E, Nathanson J, et al. The association between childhood adversity and components of metabolic syndrome in adults with mood disorders: results from the international mood disorders collaborative project. Int J Psychiatry Med 2012;43(2):165-77.

(18) Afifi TO, Mota N, MacMillan HL, Sareen J. Harsh physical punishment in childhood and adult physical health. Pediatrics 2013;132(2):e333-e340.

(19) Rich-Edwards JW, Spiegelman D, Lividoti Hibert EN, Jun HJ, Todd TJ, Kawachi I, et al. Abuse in childhood and adolescence as a predictor of type 2 diabetes in adult women. Am J Prev Med 2010;39(6):529-36.

(20) Scott KM, Von KM, Angermeyer MC, Benjet C, Bruffaerts R, de GG, et al. Association of childhood adversities and early-onset mental disorders with adult-onset chronic physical conditions. Arch Gen Psychiatry 2011;68(8):838-44.

(21) Lee C, Tsenkova V, Carr D. Childhood trauma and metabolic syndrome in men and women. Soc Sci Med 2014;105:122-30.

(22) Batten SV, Aslan M, Maciejewski PK, Mazure CM. Childhood maltreatment as a risk factor for adult cardiovascular disease and depression. J Clin Psychiatry 2004;65(2):249-54.

(23) Lissau I, Sorensen TI. Parental neglect during childhood and increased risk of obesity in young adulthood. Lancet 1994;343(8893):324-7.

(24) McEwen BS. Allostasis and allostatic load: implications for neuropsychopharmacology. Neuropsychopharmacology 2000;22(2):108-24.

(25) Power C, Pinto Pereira SM, Li L. Childhood maltreatment and BMI trajectories to mid-adult life: follow-up to age 50 y in a British birth cohort. PLoS One 2015;10(3):e0119985.

(26) Vamosi M, Heitmann BL, Kyvik KO. The relation between an adverse psychological and social environment in childhood and the development of adult obesity: a systematic literature review. Obes Rev 2010;11(3):177-84.

(27) Pinto Pereira SM, Li L, Power C. Child Maltreatment and Adult Living Standards at 50 Years. Pediatrics 2017;139(1): e20161595.

(28) Geoffroy MC, Pinto PS, Li L, Power C. Child Neglect and Maltreatment and Childhood-to-Adulthood Cognition and Mental Health in a Prospective Birth Cohort. J Am Acad Child Adolesc Psychiatry 2016;55(1):33-40.

(29) Jun HJ, Rich-Edwards JW, Boynton-Jarrett R, Austin SB, Frazier AL, Wright RJ. Child abuse and smoking among young women: the importance of severity, accumulation, and timing. J Adolesc Health 2008;43(1):55-63.

(30) Widom CS, White HR, Czaja SJ, Marmorstein NR. Long-term effects of child abuse and neglect on alcohol use and excessive drinking in middle adulthood. J Stud Alcohol Drugs 2007;68(3):317-26.

(31) Miller GE, Chen E, Parker KJ. Psychological stress in childhood and susceptibility to the chronic diseases of aging: moving toward a model of behavioral and biological mechanisms. Psychol Bull 2011;137(6):959-97.

For peer review only - http://bmjopen.bmj.com/site/about/guidelines.xhtml

For peer review only

(32) Atherton K, Fuller E, Shepherd P, Strachan DP, Power C. Loss and representativeness in a biomedical survey at age 45 years: 1958 British birth cohort. J Epidemiol Community Health 2008;62(3):216-23.

(33) Friedewald WT, Levy RI, Fredrickson DS. Estimation of the concentration of low-density lipoprotein cholesterol in plasma, without use of the preparative ultracentrifuge. Clin Chem 1972;18(6):499-502.

(34) Clinical Guidelines on the Identification, Evaluation, and Treatment of Overweight and Obesity in Adults--The Evidence Report. National Institutes of Health. Obes Res 1998;6 Suppl 2:51S-209S.

(35) Executive Summary of The Third Report of The National Cholesterol Education Program (NCEP) Expert Panel on Detection, Evaluation, And Treatment of High Blood Cholesterol In Adults (Adult Treatment Panel III). JAMA 2001;285(19):2486-97.

(36) Li L, Pinot de MA, Power C. Predicting cardiovascular disease risk factors in midadulthood from childhood body mass index: utility of different cutoffs for childhood body mass index. Am J Clin Nutr 2011;93(6):1204-11.

(37) 1999 World Health Organization-International Society of Hypertension Guidelines for the Management of Hypertension. Guidelines Subcommittee. J Hypertens 1999;17(2):151-83.

(38) Miller M, Stone NJ, Ballantyne C, Bittner V, Criqui MH, Ginsberg HN, et al. Triglycerides and cardiovascular disease: a scientific statement from the American Heart Association. Circulation 2011;123(20):2292-333.

(39) Thomas C, Hypponen E, Power C. Prenatal exposures and glucose metabolism in adulthood: are effects mediated through birth weight and adiposity? Diabetes Care 2007;30(4):918-24.

(40) Parsons TJ, Power C, Manor O. Longitudinal physical activity and diet patterns in the 1958 British Birth Cohort. Med Sci Sports Exerc 2006;38(3):547-54.

(41) Rodgers B, Pickles A, Power C, Collishaw S, Maughan B. Validity of the Malaise Inventory in general population samples. Soc Psychiatry Psychiatr Epidemiol 1999;34(6):333-41.

(42) Cole TJ. Sympercents: symmetric percentage differences on the 100 log(e) scale simplify the presentation of log transformed data. Stat Med 2000;19(22):3109-25.

(43) Tobin MD, Sheehan NA, Scurrah KJ, Burton PR. Adjusting for treatment effects in studies of quantitative traits: antihypertensive therapy and systolic blood pressure. Stat Med 2005;24(19):2911-35.

(44) Ki M, Pouliou T, Li L, Power C. Physical (in)activity over 20 y in adulthood: associations with adult lipid levels in the 1958 British birth cohort. Atherosclerosis 2011;219(1):361-7.

(45) Bennett WL, Maruthur NM, Singh S, Segal JB, Wilson LM, Chatterjee R, et al. Comparative effectiveness and safety of medications for type 2 diabetes: an update including new drugs and 2-drug combinations. Ann Intern Med 2011;154(9):602-13.

(46) Richiardi L, Bellocco R, Zugna D. Mediation analysis in epidemiology: methods, interpretation and bias. Int J Epidemiol 2013;42(5):1511-9.

(47) Nguyen QC, Osypuk TL, Schmidt NM, Glymour MM, Tchetgen Tchetgen EJ. Practical guidance for conducting mediation analysis with multiple mediators using inverse odds ratio weighting. Am J Epidemiol 2015;181(5):349-56.

For peer review only - http://bmjopen.bmj.com/site/about/guidelines.xhtml

(48) Schafer JL. Multiple imputation: a primer. Stat Methods Med Res 1999;8(1):3-15.

(49) Gilbert R, Widom CS, Browne K, Fergusson D, Webb E, Janson S. Burden and consequences of child maltreatment in high-income countries. Lancet 2009;373(9657):68-81.

(50) Denholm R, Power C, Li L. Adverse childhood experiences and child-to-adult height trajectories in the 1958 British birth cohort. Int J Epidemiol 2013;42(5):1399-409.

(51) Fergusson DM, Horwood LJ, Woodward LJ. The stability of child abuse reports: a longitudinal study of the reporting behaviour of young adults. Psychol Med 2000;30(3):529-44.

(52) Denholm R, Power C, Thomas C, Li L. Child maltreatment and household dysfunction in a British birth cohort. Child Abuse Review 2013;22:340-53.

(53) Reuben A, Moffitt TE, Caspi A, Belsky DW, Harrington H, Schroeder F, et al. Lest we forget: comparing retrospective and prospective assessments of adverse childhood experiences in the prediction of adult health. J Child Psychol Psychiatry 2016;57(10):1103-12.

(54) Wilder LB, Bachorik PS, Finney CA, Moy TF, Becker DM. The effect of fasting status on the determination of low-density and high-density lipoprotein cholesterol. Am J Med 1995;99(4):374-7.

(55) Emberson JR, Whincup PH, Walker M, Thomas M, Alberti KG. Biochemical measures in a population-based study: effect of fasting duration and time of day. Ann Clin Biochem 2002;39(Pt 5):493-501.

(56) Zweers A, Yaron E, Groen JJ. A study of fasting and postprandial serum triglycerides in connection with epidemiological surveys. Clin Chim Acta 1968;19(2):267-75.

(57) Nordestgaard BG, Benn M, Schnohr P, Tybjaerg-Hansen A. [Non-fasting triglycerides and risk of for myocardial infarction and death among women and men]. Ugeskr Laeger 2007;169(45):3865-8.

(58) Bansal S, Buring JE, Rifai N, Mora S, Sacks FM, Ridker PM. Fasting compared with nonfasting triglycerides and risk of cardiovascular events in women. JAMA 2007;298(3):309-16.

(59) Seaman SR, White IR, Copas AJ, Li L. Combining multiple imputation and inverse-probability weighting. Biometrics 2012;68(1):129-37.

(60) Kendall-Tackett K. The health effects of childhood abuse: four pathways by which abuse can influence health. Child Abuse Negl 2002;26(6-7):715-29.

(61) Friedman EM, Karlamangla AS, Gruenewald TL, Koretz B, Seeman TE. Early life adversity and adult biological risk profiles. Psychosom Med 2015;77(2):176-85.

For peer review only - http://bmjopen.bmj.com/site/about/guidelines.xhtml

Table legends:

Table 1: Childhood maltreatment and adult cardiometabolic biomarkers (mean (SD) or %)

Table 2: Associations (mean difference or OR) between childhood maltreatment and adult cardiometabolic markers unadjusted and adjusted for early-life factors

Table 3: Associations (mean difference or OR) for childhood maltreatment and adult cardiometabolic markers, adjusted for intermediary factors, separately and combined

Table 4: Associations (mean difference or OR) between number of maltreatments and cardiometabolic markers at 45y

For peer review only

Table 1: Childhood maltreatment and adult cardiometabolic biomarkers (mean(SD) or %)

	N	Males	Females
**Childhood maltreatment**			
Neglect†	8734	18.9%	16.0%
Abuse			
Physical	9309	6.0%	6.2%
Sexual	9309	0.5%	2.7%
Psychological	9310	8.3%	11.6%
**Number of types**			
0	6371	73.4%	73.5%
1	1771	21.3%	19.5%
2	384	4.1%	4.7%
≥3	150	1.2%	2.3%
**Cardiometabolic markers(45y)‡**			
BMI (kg/m^2)	9348	27.84(4.37)	27.00(5.64)
General obesity(≥30kg/m^2)		25.4%	23.7%
Waist circumference(cm)	9291	98.47(11.24)	85.59(12.93)
Central obesity(≥102/88cm)$		32.7%	36.9%
Blood pressure(mmHg)			
SBP	9297	133.33(15.35)	120.76(16.01)
DBP	9297	82.51(10.84)	76.07(10.77)
High SBP/DBP(≥140/90)	9297	34.6%	16.3%
Blood lipids(mmol/L)			
Total cholesterol	7824	6.10(1.17)	5.71(1.02)
HDL-cholesterol	7808	1.43(0.34)	1.69(0.41)
Low(≤1.0/1.3) HDL-c$	7847	11.8%	20.3%
LDL-cholesterol	7391	3.60(0.96)	3.30(0.89)
High(>4.13) LDL-c	7451	27.1%	15.8%
Triglycerides	7799	2.52(1.83)	1.60(1.14)
High(>2.3) triglycerides	7837	42.7%	16.7%
HbA_{1c}(%)	7923	5.34(0.82)	5.20(0.68)
High(>6%) HbA_{1c}	7964	6.1%	4.2%
Metabolic syndrome	7640	15.1%	10.2%

†≥2 indicators (7 and/or 11y); sample for individuals with cardiometabolic marker(s)
‡all continuous measures of cardiometabolic markers were adjusted for medication. For binary outcomes (hypertension, dyslipidemia, or T2 diabetes), those on medication were in risk groups. N differs as some with medication information but no blood sample measures were in risk group.
$cut-offs for males/females.

For peer review only - http://bmjopen.bmj.com/site/about/guidelines.xhtml

Table 2: Associations (mean difference or OR) between childhood maltreatment and adult cardiometabolic markers unadjusted and adjusted for early-life factors

	Neglect		Physical abuse		Sexual abuse		Psychological abuse	
	Model 1	Model 2	Model 1	Model 2	Model 1	Model 2	Model 1	Model 2
**Mean difference(95%CI)‡**								
BMI(kg/m²)	**0.87(0.59,1.15)**	**0.53(0.23,0.83)**	**0.83(0.39,1.27)**	**0.72(0.28,1.16)**	0.34(-0.50,1.18)	0.14(-0.70,0.97)	**0.36(0.01,0.71)**	0.28(-0.07,0.63)
Waist circumference(cm)	**1.88(1.19,2.57)**	**1.23(0.51,1.96)**	**1.44(0.38,2.50)**	**1.29(0.23,2.35)**	0.15(-1.89,2.19)	-0.16(-2.19,1.88)	**0.89(0.05,1.73)**	0.74(-0.10,1.58)
**Blood pressure**								
SBP(mmHg)	**1.40(0.51,2.29)**	0.80(-0.14,1.73)	-0.46(-1.83,0.91)	-0.83(-2.20,0.54)	-2.90(-5.56,-0.24)#	-3.50(-6.16,-0.84)#	-0.28(-1.38,0.81)	-0.45(-1.54,0.65)
DBP(mmHg)	**0.99(0.38,1.59)**	0.58(-0.06, 1.22)	0.17(-0.78,1.12)	-0.08(-1.03,0.87)	-2.02(-3.84,-0.20)#	-2.45(-4.27,-0.63)#	0.54(-0.21,1.30)	0.43(-0.32,1.19)
**Blood lipids**								
Total cholesterol(mmol/L)	0.06(-0.01,0.13)	0.04(-0.030.12)	0.08(-0.03,0.18)	0.07(-0.04,0.17)	0.16(-0.06,0.38)	0.14(-0.08,0.36)	-0.02(-0.10,0.07)	-0.02(-0.11,0.07)
HDL-c(mmol/L)					-0.07(-0.14,0.01)	-0.05(-0.12,0.02)	**-0.05(-0.07,-0.02)**	**-0.04(-0.07,-0.01)**
males	**-0.03(-0.06,0)**	-0.01(-0.04,0.02)	0.00(-0.05,0.05)	0.00(-0.05,0.05)				
females	**-0.08(-0.12,-0.05)**	**-0.05(-0.08,-0.01)**	**-0.08(-0.14,-0.02)**	**-0.06(-0.12,-0.01)**				
LDL-c	**0.06(0.01,0.12)**	0.05(-0.02,0.11)	0.07(-0.02,0.17)	0.06(-0.03,0.16)	**0.19(0,0.38)**	0.17(-0.02,0.36)	0.01 (-0.07,0.08)	0(-0.08,0.08)
Triglycerides†%	**7.7(4.3,11.1)**	**3.9(0.3,7.5)**	5.5(-0.1,1.1)	3.6(-2.0,9.2)	4.5(-6.5,15.5)	1.7(-9.1,12.5)	2.6(-1.8,7.0)	1.7(-2.7,6.1)
HbA_{1c}†%	**1.7(0.9,2.5)**	**1.2(0.4,2.0)**			**2.7(0.3,5.1)**	**2.4(0.0,4.8)**	0.6(-0.4,1.6)	0.5(-0.5,2.0)
males			**2.8(1.0,4.6)**	**2.5(0.7,4.3)**				
females			0.8(-0.8,2.4)	0.5(-1.1,2.1)				
**OR(95%CI) for elevated levels$**								
General obesity	**1.32(1.17,1.49)**	**1.16(1.02,1.32)**	**1.42(1.18,1.71)**	**1.36(1.13,1.64)**	1.20(0.84,1.73)	1.12(0.77,1.61)	**1.17(1.00,1.36)**	1.13(0.97,1.32)
Central obesity	**1.27(1.13,1.43)**	**1.15(1.02,1.30)**	**1.41(1.19,1.68)**	**1.38(1.16,1.65)**	1.01(0.72,1.42)	0.96(0.68,1.36)	1.13(0.98,1.30)	1.11(0.96,1.27)
Hypertension	**1.21(1.07,1.37)**	1.12(0.98,1.27)	1.00(0.81,1.22)	0.95(0.77,1.17)	0.46(0.26,0.80)	0.43(0.24,0.74)	1.02(0.86,1.20)	1.00(0.85,1.18)
HDL-c	**1.36(1.16,1.59)**	1.15(0.98,1.36)	**1.29(1.01,1.65)**	1.20(0.94,1.54)	1.43(0.93,2.21)	1.29(0.83,2.01)	1.12(0.91,1.37)	1.08(0.88,1.32)
LDL-c	1.09(0.94,1.25)	1.04(0.89,1.21)	**1.27(1.02,1.59)**	**1.25(1.00,1.56)**	1.46(0.92,2.31)	1.41(0.89,2.23)	1.00(0.83,1.21)	0.99(0.82,1.19)
Triglycerides	**1.19(1.04,1.36)**	1.08(0.93,1.24)	**1.26(1.02,1.57)**	1.20(0.97,1.49)	1.30(0.83,2.03)	1.20(0.77,1.88)	**1.23(1.04,1.47)**	**1.21(1.02,1.44)**
HbA_{1c}	**1.43(1.11,1.84)**	1.24(0.95,1.61)	1.38(0.91,2.11)	1.27(0.83,1.94)	1.53(0.68,3.45)	1.36(0.60,3.09)	1.20(0.84,1.73)	1.16(0.81,1.67)
Metabolic syndrome	1.22(0.99,1.50)	1.08(0.89,1.32)	1.20(0.86,1.69)	1.14(0.81,1.60)	1.48(0.48,4.53)	1.37(0.45,4.20)	1.18(0.93,1.51)	1.16(0.91,1.47)

NB: genders combined except when gender*maltreatment interaction p≤0.05. Estimates that reached significance with $P<0.05$ were bold-faced.
Model 1 includes gender and factors affecting measurement (for BP: measured room temperature; lipids and HBA1c: month of examination, time of blood collection, postal delay of blood sample, self-reported time since last meal; for females: oral contraception and HRT). For metabolic syndrome: all factors included.
Model 2: model 1 factors, self-reported family history of diabetes (for HbA1c), early-life factors (birthweight for gestational age, social class at birth, housing tenure and crowding at 7y)
†log-transformed and converted to % [NB: for HbA1c and triglycerides parameters are % of units (%)]
‡all cardiometabolic markers (continuous measures) were adjusted for medication. For binary outcomes (hypertension, dyslipidemia, or T2 diabetes), those on medication were in risk groups
$details of risk groups in **Table 1**
#included interaction term for gender (not separately by gender because of small Ns).

For peer review only - http://bmjopen.bmj.com/site/about/guidelines.xhtml

Table 3: Associations (mean difference or OR) for childhood maltreatment and adult cardiometabolic markers, adjusted for intermediary factors, separately and combined

	Model 2	Model 3 (+BMI7&45)	Model 4 (+adult SEP)	Model 5 (+lifestyle factors)	Model 6 (+depressive symptoms)	Model 7 (+all factors)
**Mean difference(95%CI)‡**						
**Neglect**						
BMI(kg/m^2)	**0.53(0.23,0.83)**		**0.30(0.0,0.61)**	**0.50(0.20,0.80)**	**0.50(0.20,0.80)**	**0.30(0.0,0.60)**
Waist circumference(cm)	**1.23(0.51,1.96)**		0.59(-0.15,1.33)	**1.09(0.37,1.81)**	**1.14(0.41,1.86)**	0.54(-0.20,1.27)
HDL-c(mmol/L)(*females*)	**-0.05(-0.08,-0.01)**	-0.03(-0.07,0.01)	-0.02(-0.06,0.02)	-0.02(-0.06,0.02)	**-0.04(-0.08,0)**	0.0(-0.03,0.03)
Triglycerides†%	**3.9(0.3,7.5)**	2.2(-1.4,5.8)	2.4(-1.4,6.2)	2.4(-1.2,6.0)	3.4(-0.2,7.0)	0.5(-3.1,4.1)
HbA$_{1c}$(%)†%	**1.2(0.4,2.0)**	**1.0(0.2,1.8)**	0.7(-0.1,1.5)	0.7(-0.1,1.5)	**1.1(0.3,1.9)**	0.4(-0.4,1.2)
**Physical abuse**						
BMI(kg/m^2)	**0.72(0.28,1.16)**		**0.71(0.27,1.14)**	**0.79(0.36,1.22)**	**0.66(0.22,1.10)**	**0.77(0.34,1.21)**
Waist circumference(cm)	**1.29(0.23,2.35)**		**1.25(0.19,2.30)**	**1.32(0.27,2.37)**	1.04(-0.02,2.10)	**1.22(0.17,2.28)**
HDL-c(mmol/L)(*females*)	**-0.06(-0.12,-0.01)**	-0.04(-0.10,0.01)	**-0.06(-0.12,-0.01)**	-0.04(-0.09,0.02)	-0.05(-0.11,0.001)	-0.01(-0.06,0.04)
HbA$_{1c}$(%)(*males*)†%	**2.5(0.7,4.3)**	**2.1(0.3, 3.9)**	**2.4(0.6,4.2)**	**1.9(0.1,3.7)**	**2.3(0.3,4.3)**	1.4(-0.4,3.2)
**Sexual abuse**						
HbA$_{1c}$(%)†%	**2.4(0.0,4.8)**	2.2(-0.2,4.6)	1.8(-0.6,4.2)	1.2(-1.2,3.6)	2.0(-0.4,4.4)	0.9(-1.5,3.3)
**Psychological abuse**						
HDL-c(mmol/L)	**-0.04(-0.07,-0.01)**	**-0.04(-0.07,-0.01)**	**-0.04(-0.07,-0.01)**	-0.02(-0.05,0.004)	-0.03(-0.06,0.001)	-0.02(-0.04,0.01)
**OR(95% CI) for elevated levels$**						
**Neglect**						
General obesity	**1.16(1.02,1.32)**		1.06(0.93,1.21)	1.13(0.99,1.29)	**1.15(1.01,1.30)**	1.05(0.92,1.20)
Central obesity	**1.15(1.02,1.30)**		1.05(0.92,1.18)	**1.13(1.00,1.27)**	**1.13(1.00,1.28)**	1.04(0.92,1.18)
**Physical abuse**						
General obesity	**1.36(1.13,1.64)**		**1.36(1.13,1.64)**	**1.38(1.14,1.66)**	**1.33(1.10,1.60)**	**1.37(1.13,1.66)**
Central obesity	**1.38(1.16,1.65)**		**1.38(1.16,1.64)**	**1.39(1.17,1.66)**	**1.34(1.12,1.60)**	**1.38(1.16,1.65)**
LDL-c	**1.25(1.00,1.56)**	1.21(0.96,1.51)	1.24(0.99,1.56)	1.16(0.93,1.46)	1.21(0.96,1.51)	1.09(0.87,1.37)
**Sexual abuse**						
LDL-c	1.41(0.89,2.23)	1.41(0.88,2.24)	1.38(0.87,2.19)	1.26(0.79,2.00)	1.34(0.84,2.13)	1.23(0.76,1.98)
**Psychological abuse**						
Triglycerides	1.21(1.02,1.44)	1.22(1.02,1.47)	1.21(1.02,1.44)	1.18(0.99,1.40)	1.15(0.96,1.37)	1.14(0.95,1.37)

NB: associations at P<0.05 (or borderline for sexual abuse) in Model 2, **Table 2** included here. Estimates that reached significance with P<0.05 were bold-faced.
Genders combined except when gender*maltreatment interaction P<0.05.
Model 2: model 1 factors, self-reported family history of diabetes (for HbA$_{1c}$), early-life factors (see **Table 2** footnotes)
Model 3: model 2 factors, measured BMI (7 and 45y).
Model 4: model 2 factors, adult social class (SEP) (own occupation at 42y (33y if missing), qualifications by 33y).

For peer review only - http://bmjopen.bmj.com/site/about/guidelines.xhtml

Model 5: model 2 factors, lifestyles at 42y (smoking, alcohol consumption, physical activity).
Model 6: model 2 factors, depressive symptoms at 42y (0-15 Malaise Inventory items).
Model 7: all variables.
†log-transformed and converted to % [NB: for HbA_{1c} and triglycerides parameters are % of units (%)]
‡all cardiometabolic markers (continuous measures) were adjusted for medication. For binary outcomes (hypertension, dyslipidemia, or T2 diabetes), those on medication were in risk groups
$details of risk groups in **Table 1**

For peer review only

Table 4 Associations (mean difference or OR) between number of maltreatments and cardiometabolic markers at 45y

	Number of maltreatments Per increase#
**Mean difference (95%CI)‡**	
BMI (kg/m²)	**0.33(0.17,0.49)**
Waist circumference (cm)	
Males	0.23(-0.33,0.79)
Females	**1.14(0.57,1.71)**
Blood pressure	
SBP mmHg	-0.10(-0.61,0.41)
DBP mmHg	0.18(-0.17,0.53)
Blood lipids	
Total cholesterol mmol/L	0.02(-0.02,0.06)
HDL-c mmol/L	
Males	-0.01(-0.03,0.01)
Females	**-0.04(-0.06,-0.02)**
LDL-c	0.03(-0.01,0.07)
Triglycerides† %	**2.2(0.0,4.4)**
HbA_{1c} † %	**0.8(0.4,1.3)**
**OR (95%CI) for elevated levels$**	
General obesity	**1.13(1.05,1.21)**
Central obesity	**1.12(1.05,1.20)**
Hypertension	1.01(0.94,1.09)
HDL-c	**1.11(1.01,1.21)**
LDL-c	1.05(0.97,1.15)
Triglycerides	**1.10(1.01,1.20)**
HbA_{1c}	1.16(0.99,1.36)
Metabolic syndrome	1.10(0.96,1.26)

NB: analyses are for genders combined except where p≤0.05 for gender*maltreatment interaction where analyses are for males and females separately. Estimates that reached significance with P<0.05 were bold-faced.
Mean difference or OR estimated from model adjusted for early life factors, including gender and factors affecting measurement (for BP: measured room temperature; lipids and HBA1c: examination month, time of blood collection, postal delay of blood sample, time since last meal; for females: oral contraception and HRT), plus self-reported family history of diabetes (for HbA_{1c}, diabetes) and early-life factors, including birthweight for gestational age, social class at birth, housing tenure and crowding at 7y
†log transformed and converted to % [NB: for HbA_{1c} the parameters are % of the units (%)]
‡all cardiometabolic markers (continuous measures) were adjusted for medication. For binary outcomes (hypertension, dyslipidemia, or T2 diabetes), those on medication were in risk groups
$details of risk groups in **Table 1**
See also Table S3

For peer review only

For peer review only - http://bmjopen.bmj.com/site/about/guidelines.xhtml

Supplemental Table S1: Definition of child maltreatment and representative variables from the 1958 British birth cohort.

	Definition(44)	1958 British birth cohort variables*	Reference age	Age collected (Ascertainment method¥)
Neglect (prospective)‡	Failure to meet a child's basic physical, emotional, medical/dental, or education need; failure to provide adequate nutrition, hygiene, or shelter; or failure to ensure a child's safety	constructed from:<br>- child looks undernourished, scruffy or dirty<br>- hardly ever takes outings with mother<br>- hardly ever takes outings with father<br>- mother has little interest in education<br>- father has little interest in education	 7y, 11y<br>7y, 11y<br>7y, 11y<br>7y, 11y<br>7y, 11y	 7y, 11y(T)<br>7y, 11y(P)<br>7y, 11y(P)<br>7y, 11y(T)<br>7y, 11y(T)
Sexual abuse	Any completed or attempted sexual act, sexual contact, or non-contact sexual interaction with a child by a caregiver.	- I was sexually abused by a parent	0-16y	45y(S)
Physical abuse	Intentional use of physical force or implements against a child that results in, or has the potential to result in, physical injury.	- I was physically abused by a parent – punched, kicked or hit or beaten with an object, or needed medical treatment	0-16y	45y(S)
Psychological abuse	Intentional behaviour that conveys to a child that h/she is worthless, flawed, unloved, unwanted, endangered, or valued only in meeting another's needs.<br>*UK definition includes harmful (unintentional) parent-child interactions: 'the persistent emotional ill-treatment of a child such as to cause severe and persistent adverse effects on the child's emotional development'*	- I was verbally abused by a parent<br>- I suffered humiliation, ridicule, bullying or mental cruelty from a parent	0-16y	45y(S)

*for retrospective reports at 45y, information was obtained via CASI (direct computer data entry) derived from the Personality and Total Health Through Life Project (50), details of which are provided elsewhere(25). Participants were instructed: "The following are statements about your childhood. For each, please say whether the statement applies to you." Response options were: "Yes", "No", "Can't say".

¥ (S): self-report; (T): teacher-report; (P): parent-report

‡neglect (prospective) defined as ≥2 items at either 7 or 11y (note at each age, if one or two items were missing we used the sum of the remaining 4 or 3 items respectively; if >2 items were missing, we treated neglect as missing)

For peer review only

For peer review only - http://bmjopen.bmj.com/site/about/guidelines.xhtml

Supplemental Table S2 Associations (mean difference or OR) between childhood maltreatment and cardiometabolic biomarkers at 45y – adjusted for lifestyle factors individually and jointly

**Mean difference (95%CI)‡**	Model 2	+adult lifestyle factors˜	+smoking only	+alcohol consumption only	+physical activity only
**Neglect**					
BMI (kg/m²)	**0.53(0.23,0.83)**	**0.50(0.20,0.80)**	**0.60(0.31,0.90)**	**0.42(0.13,0.72)**	**0.51(0.21,0.81)**
Waist circumference (cm)	**1.23(0.51,1.96)**	**1.09(0.37,1.81)**	**1.30(0.58,2.03)**	**1.03(0.30,1.75)**	**1.18(0.46,1.90)**
HDL-c (mmol/L) (females)	**-0.05(-0.08,-0.01)**	-0.02(-0.06,0.02)	-0.03(-0.07,0.004)	-0.03(-0.07,0.004)	-0.05(-0.08,0.01)
Triglycerides† %	**3.9(0.3,7.5)**	2.4(-1.2,6.0)	2.8(-0.8,6.4)	3.5(-0.1,7.1)	**3.8(0.2,7.4)**
HbA_{1c}(%)† %	**1.2(0.4,2.0)**	0.7(-0.1,1.5)	**0.9(0.1,1.7)**	**1.0(0.2,1.8)**	**1.2(0.4,2.0)**
**Physical abuse**					
BMI (kg/m²)	**0.72(0.28,1.16)**	**0.79(0.36,1.22)**	**0.83(0.39,1.27)**	**0.65(0.21,1.08)**	**0.73(0.30,1.17)**
Waist circumference (cm)	**1.29(0.23,2.35)**	**1.32(0.27,2.37)**	**1.38(0.32,2.44)**	**1.13(0.09,2.19)**	**1.32(0.27,2.37)**
HDL-c (mmol/L) (females)	**-0.06(-0.12,-0.01)**	-0.04(-0.09,0.02)	-0.05(-0.10,0.01)	**-0.05(-0.12,-0.01)**	**-0.06(-0.12,-0.01)**
HbA_{1c} (%) (males)† %	**2.5(0.7,4.3)**	**1.9(0.1,3.7)**	**1.9(0.1,3.7)**	**2.3(0.5,4.1)**	**2.5(0.7,4.3)**
**Sexual abuse**					
HbA_{1c}(%)† %	**2.4(0.0,4.8)**	1.2(-1.2,3.6)	1.6(-0.8,4.0)	1.9(-0.5,4.3)	2.2(-0..2,4.6)
**Psychological abuse**					
HDL-c (mmol/L)	**-0.04(-0.07,-0.01)**	-0.02(-0.05,0.004)	**-0.03(-0.06,-0.005)**	-0.03(-0.06,0.001)	**-0.04(-0.07,-0.013)**
**OR (95%CI) for elevated levels$**					
**Neglect**					
General obesity	**1.16(1.02,1.32)**	1.13(0.99,1.29)	**1.18(1.03,1.34)**	1.11(0.98,1.27)	**1.15(1.01,1.31)**
Central obesity	**1.15(1.02,1.30)**	**1.13(1.00,1.27)**	**1.16(1.03,1.31)**	**1.12(0.99,1.26)**	**1.14(1.01,1.29)**
**Physical abuse**					
General obesity	**1.36(1.13,1.64)**	**1.38(1.14,1.66)**	**1.39(1.15,1.68)**	**1.32(1.10,1.60)**	**1.37(1.14,1.65)**
Central obesity	**1.38(1.16,1.65)**	**1.39(1.17,1.66)**	**1.40(1.18,1.67)**	**1.36(1.14,1.62)**	**1.39(1.17,1.66)**
LDL-c	**1.25(1.00,1.56)**	1.16(0.93,1.46)	1.16(0.93,1.46)	1.24(0.99,1.55)	**1.25(1.00,1.57)**
**Sexual abuse**					
LDL-c	1.41(0.89,2.23)	1.26(0.79,2.00)	1.28(0.80,2.03)	1.39(0.87,2.20)	1.40(0.88,2.22)
**Psychological abuse**					
Triglycerides	**1.21(1.02,1.44)**	1.18(0.99,1.40)	1.18(0.99,1.40)	**1.20(1.01,1.43)**	**1.22(1.03,1.45)**

All cardiometabolic markers (continuous measures) were adjusted for medication. For binary outcomes, those on medication were in risk groups. Estimates that reached significance with *P*<0.05 were bold-faced.

Model 2 includes all in model 1, plus self-reported family history of diabetes (for HbA_{1c}, diabetes) and early-life factors (birthweight for gestational age, social class at birth, housing tenure and crowding at 7y).

†log transformed and converted to %

‡all cardiometabolic markers (continuous measures) were adjusted for medication. For binary outcomes (dyslipidemia), those on medication were in risk groups

˜shown in Table 3 as model 5: includes all in model 2, plus lifestyle factors at 42y (smoking status, alcohol consumption and physical activity).

For peer review only

Supplemental Table S3 Associations (mean difference or OR) for multiple types of maltreatment and cardiometabolic markers at 45y: unadjusted and adjusted for early-life factors

		**Number of maltreatments**						
	**0**	**1**		**2**		**≥3**		Per increase[#]
**Mean difference (95%CI)‡**		Model 1	Model 2	Model 1	Model 2	Model 1	Model 2	
BMI (kg/m^2)	-	**0.74(0.47,1.01)**	**0.47(0.19,0.74)**	**0.87(0.35,1.38)**	**0.66(0.15,1.17)**	**0.91(0.09,1.74)**	0.65(-0.18,1.47)	**0.33(0.17,0.49)**
Waist circumference (cm)								
males	-	0.74(-0.10,1.58)	0.29(-0.56,1.14)	1.13(-0.60,2.86)	0.88(-0.83,2.60)	-0.09(-3.20,3.02)	-0.46(-3.55,2.63)	0.23(-0.33,0.79)
females	-	**2.74(1.74,3.73)**	**2.19(1.17,3.21)**	**2.47(0.63,4.30)**	**2.00(0.18,3.82)**	2.32(-0.26,4.90)	1.84(-0.75,4.43)	**1.14(0.57,1.71)**
Blood pressure								
SBP mmHg	-	**1.22(0.38,2.06)**	0.70(-0.18,1.57)	-0.34(-1.96,1.28)	-0.76(-2.37,0.86)	-0.81(-3.35,1.73)	-1.53(-4.07,1.01)	-0.10(-0.61,0.41)
DBP mmHg	-	**1.01(0.43,1.59)**	**0.67(0.08,1.27)**	0.42(-0.69,1.53)	0.15(-0.96,1.25)	0.01(-1.75,1.77)	-0.45(-2.21,1.31)	0.18(-0.17,0.53)
Blood lipids								
Total cholesterol mmol/L	-	**0.06(0,0.13)**	0.05(-0.02,0.11)	0.01(-0.12,0.14)	0.01(-0.12,0.14)	0.08(-0.12,0.28)	0.07(-0.14,0.27)	0.02(-0.02,0.06)
**HDL-c mmol/L**								
**males**	-	-0.02(-0.05,0.01)	**-0.003(-0.031,0.025)**	-0.03(-0.09,0.02)	-0.02(-0.08,0.04)	-0.04(-0.15,0.07)	-0.03(-0.14,0.08)	-0.01(-0.03,0.01)
**females**	-	-0.08(-0.11,0.05)	**-0.05(-0.09,-0.02)**	-0.09(-0.15,0.02)	**-0.06(-0.13,0.0)**	-0.12(-0.21,0.03)	**-0.09(-0.18,0.0)**	**-0.04(-0.06,-0.02)**
LDL-c	-	**0.07(0.01,0.13)**	**0.06(0,0.11)**	0.04(-0.08,0.15)	0.02(-0.09,0.14)	0.09(-0.08,0.27)	0.08(-0.10,0.25)	0.03(-0.01,0.07)
Triglycerides† %	-	**6.3(2.9,9.7)**	**3.2(0,6.6)**	4.5(-1.9,10.9)	1.9(-4.5,8.3)	**10.8(0.6,21.0)**	7.4(-2.6,17.4)	**2.2(0.0,4.4)**
HbA_{1c}† %	-	**1.8(1,2.6)**	**1.4(0.6,2.2)**	**2.1(0.7,3.5)**	**1.7(0.3,3.1)**	**1.0(-1.4,3.4)**	0.5(-1.9,2.9)	**0.8(0.4,1.3)**
**OR (95%CI) for elevated levels$**								
General obesity	-	**1.29(1.15,1.45)**	**1.19(1.03,1.37)**	**1.39(1.11,1.73)**	**1.33(1.03,1.72)**	**1.45(1.03,2.05)**	1.37(0.93,2.00)	**1.13(1.05,1.21)**
Central obesity	-	**1.25(1.12,1.39)**	**1.16(1.04,1.29)**	**1.39(1.13,1.71)**	**1.32(1.07,1.62)**	1.26(0.91,1.74)	1.18(0.85,1.64)	**1.12(1.05,1.20)**
Hypertension	-	**1.18(1.06,1.34)**	1.11(0.98,1.26)	1.02(0.81,1.30)	0.97(0.76,1.23)	0.91(0.61,1.36)	0.84(0.56,1.25)	1.01(0.94,1.09)
HDL-c	-	**1.29(1.11,1.50)**	1.14(0.97,1.33)	**1.56(1.18,2.07)**	**1.41(1.06,1.87)**	1.17(0.74,1.85)	1.02(0.65,1.62)	**1.11(1.01,1.21)**
LDL-c	-	1.10(0.96,1.25)	1.06(0.92,1.22)	1.17(0.89,1.54)	1.14(0.86,1.50)	1.16(0.76,1.78)	1.12(0.73,1.72)	1.05(0.97,1.15)
Triglycerides	-	**1.24(1.09,1.40)**	**1.14(1.00,1.30)**	1.24(0.96,1.59)	1.16(0.90,1.50)	1.41(0.94,2.10)	1.30(0.87,1.94)	**1.10(1.01,1.20)**
HbA_{1c}	-	**1.47(1.15,1.87)**	**1.31(1.01,1.69)**	1.47(0.89,2.43)	1.33(0.80,2.21)	1.30(0.54,3.18)	1.12(0.46,2.74)	1.16(0.99,1.36)
Metabolic syndrome	-	**1.26(1.03,1.54)**	1.15(0.95,1.39)	1.37(0.94,1.99)	1.27(0.87,1.83)	1.15(0.61,2.16)	1.06(0.57,1.96)	1.10(0.96,1.26)

NB: analyses are for genders combined except where $p \leq 0.05$ for gender*maltreatment interaction where analyses are for males and females separately. Estimates that reached significance with $P<0.05$ were bold-faced.

[#] per increase based on Model 2

Model 1 includes gender and factors affecting measurement (measured room temperature for BP; month of examination, time of day of blood collection, postal delay of blood sample, time since last meal for lipids and HbA_{1c}; for females oral contraception and HRT for all outcomes). All factors were included for metabolic syndrome

Model 2 includes all in model 1, plus self-reported family history of diabetes (for HbA_{1c}, diabetes) and early-life factors, including birthweight for gestational age, social class at birth, housing tenure and crowding at 7y
†log transformed and converted to % [NB: for HbA_{1c} the parameters are % of the units (%)]
‡all cardiometabolic markers (continuous measures) were adjusted for medication. For binary outcomes (hypertension, dyslipidemia, or T2 diabetes), those on medication were in risk groups
$details of risk groups in **Table 1**

For peer review only - http://bmjopen.bmj.com/site/about/guidelines.xhtml

STROBE Statement—Checklist of items that should be included in reports of ***cohort studies***

	Item No	Recommendation	Reported on page #
**Title and abstract**	1	(*a*) Indicate the study's design with a commonly used term in the title or the abstract	Pages 1, 2
		(*b*) Provide in the abstract an informative and balanced summary of what was done and what was found	Page 2
**Introduction**			
Background/rationale	2	Explain the scientific background and rationale for the investigation being reported	Pages 4, 5
Objectives	3	State specific objectives, including any prespecified hypotheses	Page 5
**Methods**			
Study design	4	Present key elements of study design early in the paper	Page 7
Setting	5	Describe the setting, locations, and relevant dates, including periods of recruitment, exposure, follow-up, and data collection	Page 7
Participants	6	(*a*) Give the eligibility criteria, and the sources and methods of selection of participants. Describe methods of follow-up	Page 7
		(*b*) For matched studies, give matching criteria and number of exposed and unexposed	NA
Variables	7	Clearly define all outcomes, exposures, predictors, potential confounders, and effect modifiers. Give diagnostic criteria, if applicable	Pages 7-9
Data sources/ measurement	8*	For each variable of interest, give sources of data and details of methods of assessment (measurement). Describe comparability of assessment methods if there is more than one group	Pages 7-9
Bias	9	Describe any efforts to address potential sources of bias	Pages 10, 11
Study size	10	Explain how the study size was arrived at	Page 11
Quantitative variables	11	Explain how quantitative variables were handled in the analyses. If applicable, describe which groupings were chosen and why	Pages 10, 11
Statistical methods	12	(*a*) Describe all statistical methods, including those used to control for confounding	Pages 9-11
		(*b*) Describe any methods used to examine subgroups and interactions	NA
		(*c*) Explain how missing data were addressed	Page 11
		(*d*) If applicable, explain how loss to follow-up was addressed	Page 11
		(*e*) Describe any sensitivity analyses	Pages 10, 11
**Results**			
Participants	13*	(a) Report numbers of individuals at each stage of study—eg numbers potentially eligible, examined for eligibility, confirmed eligible, included in the study, completing follow-up, and analysed	Page 11
		(b) Give reasons for non-participation at each stage	Page 11

For peer review only - http://bmjopen.bmj.com/site/about/guidelines.xhtml

		(c) Consider use of a flow diagram	NA
Descriptive data	14*	(a) Give characteristics of study participants (eg demographic, clinical, social) and information on exposures and potential confounders	Page 10, Table 1
		(b) Indicate number of participants with missing data for each variable of interest	Page 11, Table 1
		(c) Summarise follow-up time (eg, average and total amount)	Page 7
Outcome data	15*	Report numbers of outcome events or summary measures over time	Table 1
Main results	16	(*a*) Give unadjusted estimates and, if applicable, confounder-adjusted estimates and their precision (eg, 95% confidence interval). Make clear which confounders were adjusted for and why they were included	Page 9, Tables 2-4
		(*b*) Report category boundaries when continuous variables were categorized	Pages 7-9
		(*c*) If relevant, consider translating estimates of relative risk into absolute risk for a meaningful time period	Pages 11, 12
Other analyses	17	Report other analyses done—eg analyses of subgroups and interactions, and sensitivity analyses	Pages 10, 11
**Discussion**			
Key results	18	Summarise key results with reference to study objectives	Page 13
Limitations	19	Discuss limitations of the study, taking into account sources of potential bias or imprecision. Discuss both direction and magnitude of any potential bias	Pages 13, 14
Interpretation	20	Give a cautious overall interpretation of results considering objectives, limitations, multiplicity of analyses, results from similar studies, and other relevant evidence	Pages 14-16
Generalisability	21	Discuss the generalisability (external validity) of the study results	Page 16
**Other information**			
Funding	22	Give the source of funding and the role of the funders for the present study and, if applicable, for the original study on which the present article is based	Pages 17, 18

*Give information separately for exposed and unexposed groups.

Note: An Explanation and Elaboration article discusses each checklist item and gives methodological background and published examples of transparent reporting. The STROBE checklist is best used in conjunction with this article (freely available on the Web sites of PLoS Medicine at http://www.plosmedicine.org/, Annals of Internal Medicine at http://www.annals.org/, and Epidemiology at http://www.epidem.com/). Information on the STROBE Initiative is available at http://www.strobe-statement.org.

For peer review only
